# From waste to health-supporting molecules: biosynthesis of natural products from lignin-, plastic- and seaweed-based monomers using metabolically engineered *Streptomyces lividans*

**DOI:** 10.1186/s12934-023-02266-0

**Published:** 2023-12-19

**Authors:** Kyoyoung Seo, Wei Shu, Christian Rückert-Reed, Patrick Gerlinger, Tobias J. Erb, Jörn Kalinowski, Christoph Wittmann

**Affiliations:** 1 Institute of Systems Biotechnology, Saarland University, Saarbrücken, Germany; 2 Cebitec, University of Bielefeld, Bielefeld, Germany; 3 Max Planck Institute for Terrestrial Microbiology, Marburg, Germany

**Keywords:** *Streptomyces lividans*, Renewable feedstock, Lignin, Polystyrene, Seaweed, 4-hydroxybenzoate, Protocatechuate, Mannitol, Ethylmalonyl-CoA pathway, Bottromycin, Pamamycin, Natural product

## Abstract

**Background:**

Transforming waste and nonfood materials into bulk biofuels and chemicals represents a major stride in creating a sustainable bioindustry to optimize the use of resources while reducing environmental footprint. However, despite these advancements, the production of high-value natural products often continues to depend on the use of first-generation substrates, underscoring the intricate processes and specific requirements of their biosyntheses. This is also true for *Streptomyces lividans*, a renowned host organism celebrated for its capacity to produce a wide array of natural products, which is attributed to its genetic versatility and potent secondary metabolic activity. Given this context, it becomes imperative to assess and optimize this microorganism for the synthesis of natural products specifically from waste and nonfood substrates.

**Results:**

We metabolically engineered *S. lividans* to heterologously produce the ribosomally synthesized and posttranslationally modified peptide bottromycin, as well as the polyketide pamamycin. The modified strains successfully produced these compounds using waste and nonfood model substrates such as protocatechuate (derived from lignin), 4-hydroxybenzoate (sourced from plastic waste), and mannitol (from seaweed). Comprehensive transcriptomic and metabolomic analyses offered insights into how these substrates influenced the cellular metabolism of *S. lividans*. In terms of production efficiency, *S. lividans* showed remarkable tolerance, especially in a fed-batch process using a mineral medium containing the toxic aromatic 4-hydroxybenzoate, which led to enhanced and highly selective bottromycin production. Additionally, the strain generated a unique spectrum of pamamycins when cultured in mannitol-rich seaweed extract with no additional nutrients.

**Conclusion:**

Our study showcases the successful production of high-value natural products based on the use of varied waste and nonfood raw materials, circumventing the reliance on costly, food-competing resources. *S. lividans* exhibited remarkable adaptability and resilience when grown on these diverse substrates. When cultured on aromatic compounds, it displayed a distinct array of intracellular CoA esters, presenting promising avenues for polyketide production. Future research could be focused on enhancing *S. lividans* substrate utilization pathways to process the intricate mixtures commonly found in waste and nonfood sources more efficiently.

**Supplementary Information:**

The online version contains supplementary material available at 10.1186/s12934-023-02266-0.

## Background

Natural products have long been celebrated for their vast therapeutic and commercial potential, and numerous pharmaceuticals, cosmetics, and other beneficial compounds are derived from them. Today, these products are often synthesized by microbes from agricultural feedstocks [1, 2], which demand extensive land use that competes with food production. Typical food-competing raw materials used today are glucose, starch, protein-rich meals from soybean and casein hydrolysate, yeast extract, and different peptones [3–6]. As the world grapples with burgeoning populations and diminishing availability of arable land, a reliance on land-intensive, food-based raw materials for natural product synthesis becomes increasingly unsustainable, and a shift towards more eco-conscious alternatives is needed [7, 8]. Recent prominent studies have shown that petroleum-based polystyrene waste, exhibiting a recovery rate of less than 1% due to recalcitrance [9], underutilized lignin side streams from the pulp and paper industry [10] and (often simply disposed) seaweed residuals from ocean farming [11–13] can be converted into bulk biofuels and chemicals [14–16]. This underscores the potential to use these third-generation renewables for natural product synthesis.

*Streptomyces lividans* is a prominent filamentous bacterium in the field of natural product synthesis due to its remarkable genetic manipulability and robust secondary metabolic pathways [17]. For instance, *S. lividans* TK24 has been instrumental in the derivation of heterologous producers for tunicamycin, griseorhodin, and deoxycoformycin [18] and is regarded as a promising nonmodel bacterial chassis for secondary metabolite production [19]. The recently created derivatives *S. lividans* ΔYA8 and ΔYA9 stand out in terms of their unique genetic makeup; they lack native pathways for natural product synthesis, making them ideal blank slates for the heterologous expression of exogenous biosynthetic pathways [18]. As is typical in the field, previously, production was reliant on first-generation substrates such as glucose, starch, yeast extract, and soytone [20, 21].

In terms of the valorisation of next-generation raw materials, the microbe has a broad pathway repertoire available in its genome [22]. In the context of the use of plastic waste- and lignin-based aromatic monomers, *S. lividans* possesses the β-ketoadipate pathway, a central route for aromatic catabolism, but it has not been studied experimentally for growth on these substrates. On the other hand, although not specifically tested for *S. lividans* before, mannitol, a major sugar in different seaweed hydrolysates [16], appears to be a generally well-accepted substrate for *Streptomyces* [23–26].

**Fig. 1:**
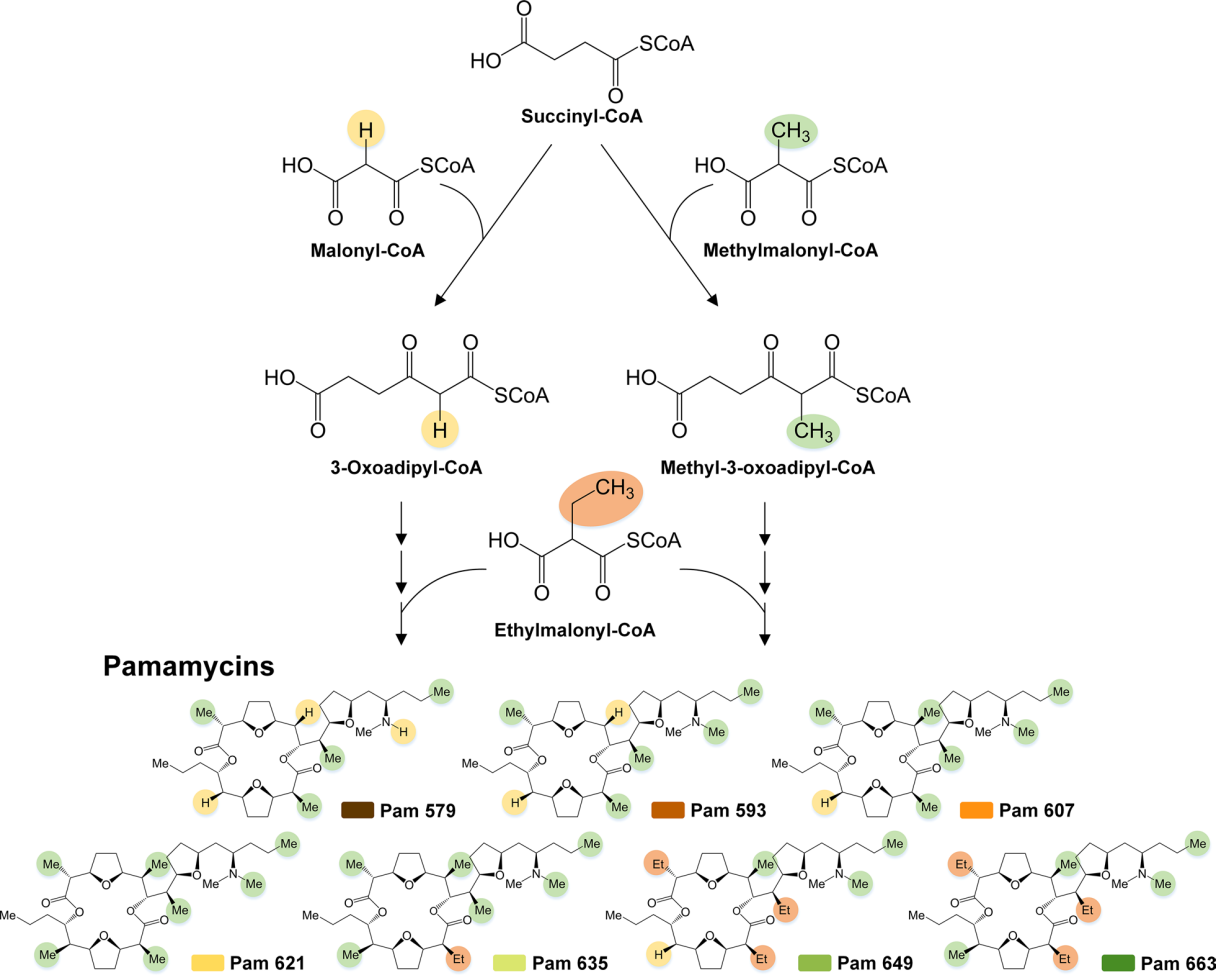
Microbial synthesis of different pamamycin derivatives. Pamamycin is biosynthetically synthesized from 4 different CoA thioesters, namely, succinyl-CoA, malonyl-CoA, methylmalonyl-CoA, and ethylmalonyl-CoA [38]. The promiscuous use of malonyl-CoA, methylmalonyl-CoA, and ethylmalonyl-CoA during the synthetic process leads to the incorporation of different side chains and the creation of pamamycin derivatives of different molecular weights, typically in the range between 579 and 663 Da

Therefore, in this work, we harnessed the capabilities of *S. lividans* to produce secondary metabolites from waste- and nonfood-based monomers. On the raw material side, we selected mannitol, a major sugar from seaweed hydrolysates [16, 27, 28], protocatechuate and 4-hydroxybenzoate, representing lignin hydrolysates [29–32], whereby 4-hydroxybenzoate exhibited additional relevance as an intermediate accessible from processed polystyrene waste [14]. On the product side, we selected the ribosomally synthesized and posttranslationally modified peptide bottromycin and the polyketide family of pamamycins, representing two major classes of natural products. Bottromycin exhibits antimicrobial activity against gram-positive pathogens [33, 34]. Its biosynthesis requires the supply of 14 amino acids to form the 7 different core and 36 follower peptides involved [35]. Pamamycins are macrolide polyketides with pronounced anti-insecticidal activity [36, 37] and are synthetized from CoA-ester precursors, including succinyl-CoA, malonyl-CoA, methylmalonyl-CoA, and ethylmalonyl-CoA [38, 39] (Fig. 1). After genomic insertion of the 18 kb bottromycin gene cluster and the 25 kb pamamycin gene cluster in *S. lividans* ΔYA8, production in the created heterologous producers was studied in batch cultures. Subsequently, comparative transcriptomics and metabolomics analysis of recombinant *S. lividans* provided a comprehensive systems view of the different waste substrates during strain growth. Finally, we demonstrated the production of bottromycin in a fed-batch process and carried out pamamycin synthesis from a hydrolysate of brown seaweed through *Himanthalia elongata*, both involving lean mineral media with exclusively next-generation carbon sources and minimized raw material pretreatment.

## Results

### The genome-minimized derivative *S. lividans* ΔYA8-DG2 produces bottromycin A2 from waste- and nonfood-based monomers

In the first step, we verified the principal capability of *S. lividans* to use sustainable monomers. We incubated *S. lividans* TK24 on solid minimal media that contained mannitol, protocatechuate or 4-hydroxybenzoate as the sole source of carbon. The strain grew well on all substrates, providing important proof of principle (Additional file 1: Fig. S1).

**Fig. 2:**
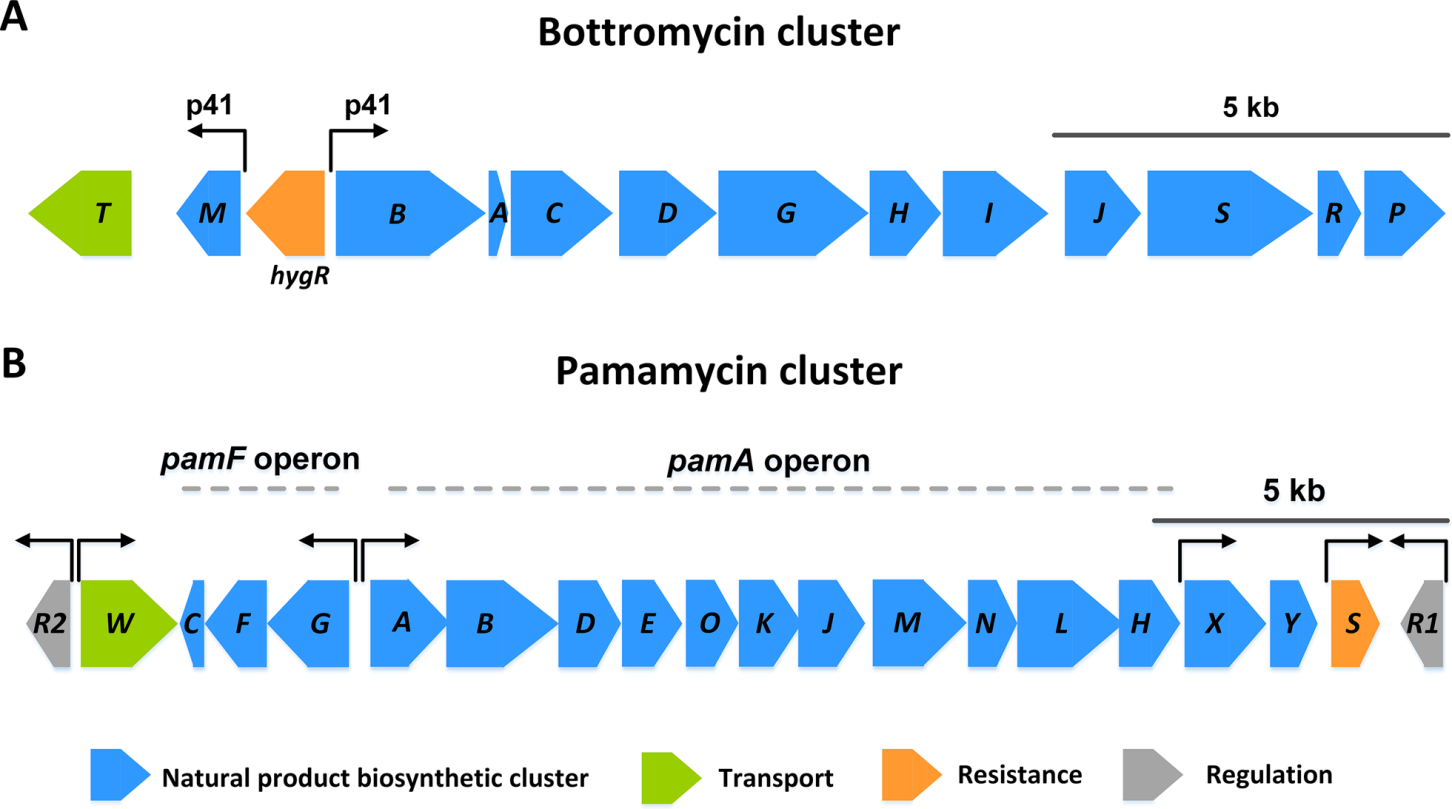
Genetic cluster architecture for the synthesis of bottromycin (**A**) and pamamycin (**B**) in recombinant *S. lividans* ΔYA8. The heterologous host *S. lividans* ΔYA8-DG2 contains a genomic copy of the 18 kb bottromycin biosynthesis-related cluster from *Streptomyces* sp. BC16019 [20]. The cluster is expressed under the control of synthetic P_41_ promoters, whereby *hygR* displays a resistance marker introduced during the cloning process. The heterologous host *S. lividans* ΔYA8-R2 contains a genomic copy of the 25 kb pamamycin biosynthesis-related cluster from *Streptomyces alboniger* DSMZ 40043 [38]. The cluster is expressed under the control of native promoters

**Fig. 3:**
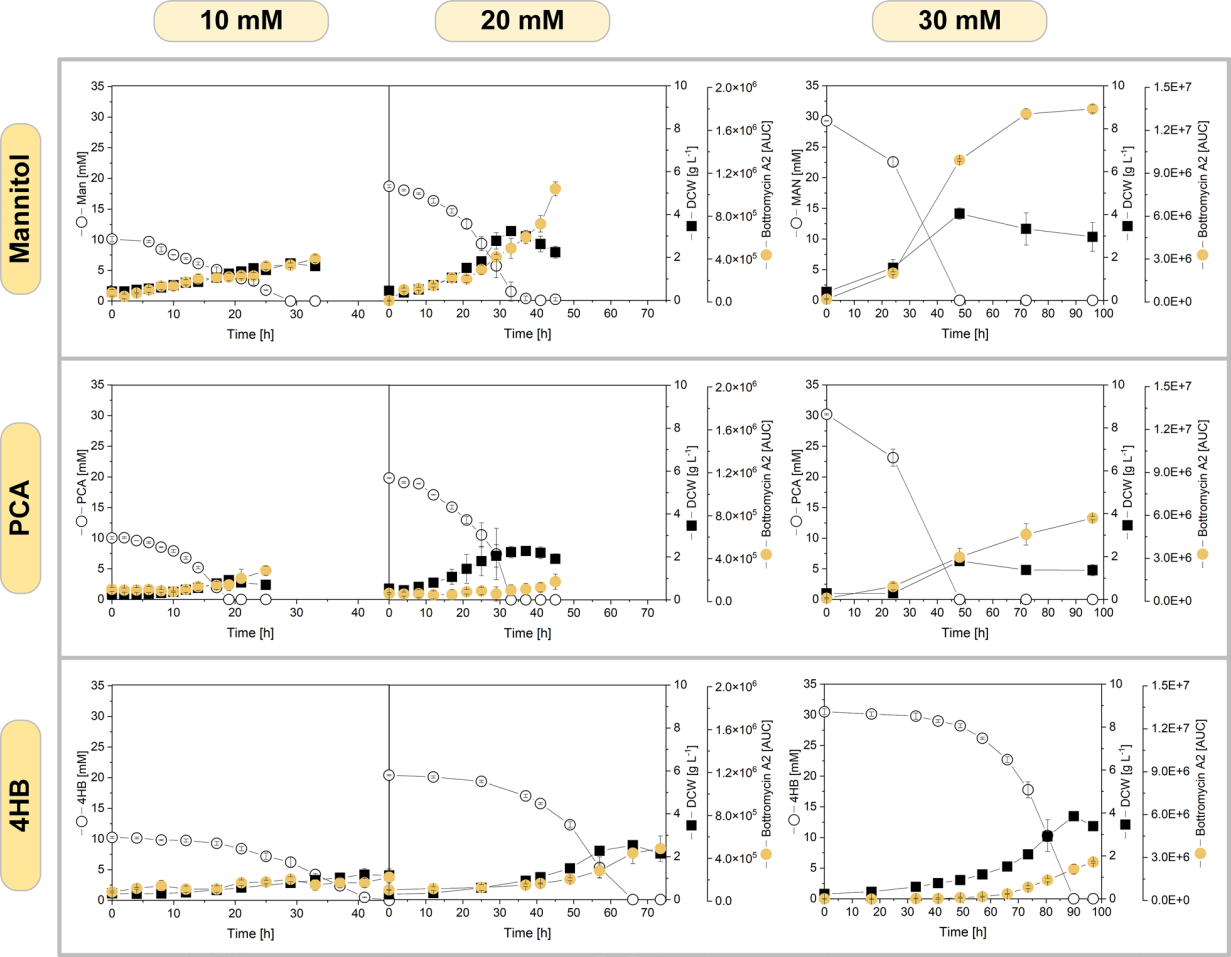
Impact of the carbon source on the growth and bottromycin A2 production of *S. lividans* ΔYA8-DG2. The recombinant strain was grown in minimal medium containing different levels of mannitol (**A**–**C**), protocatechuate (PCA) (**D**–**F**), and 4-hydroxybenzoate (4HB) (**G**–**I**). The data represent mean values and standard deviations from three biological replicates (n = 3). A minor fraction of the methylated derivative was formed in addition to bottromycin A2 (Additional file 2)

To selectively produce the heterologous natural products of interest, we utilized *S. lividans* ΔYA8, which lacks eight native biosynthesis-related gene clusters [18], as the chassis strain. It was transformed using conjugation with the cosmid DG2-km-P41hyg, which contains the bottromycin gene cluster regulated by a bidirectional pair of synthetic *P41* promoters [20]. The cluster was integrated at the *attB* site (locus tag, SLIV19310) with the Int-phiC31 recombinase (Fig. 2A). After confirming the modification through PCR and sequencing, we named the modified strain *S. lividans* ΔYA8-DG2. For production assessment in shake flask cultures, we maintained the minimal nutrient composition used in preliminary growth experiments. Using mannitol concentrations of 10, 20, and 30 mM as the sole carbon source, *S. lividans* ΔYA8-DG2 successfully synthesized bottromycin (Fig. 3A–C). Cells grew without a lag phase, and higher biomass production correlated with increased mannitol concentrations. The 30 mM mannitol cultures achieved peak bottromycin levels. The mass spectrometric analysis revealed that methylated bottromycin A2 was formed in addition to bottromycin A2 as the major derivative (Additional file 2).

**Fig. 4:**
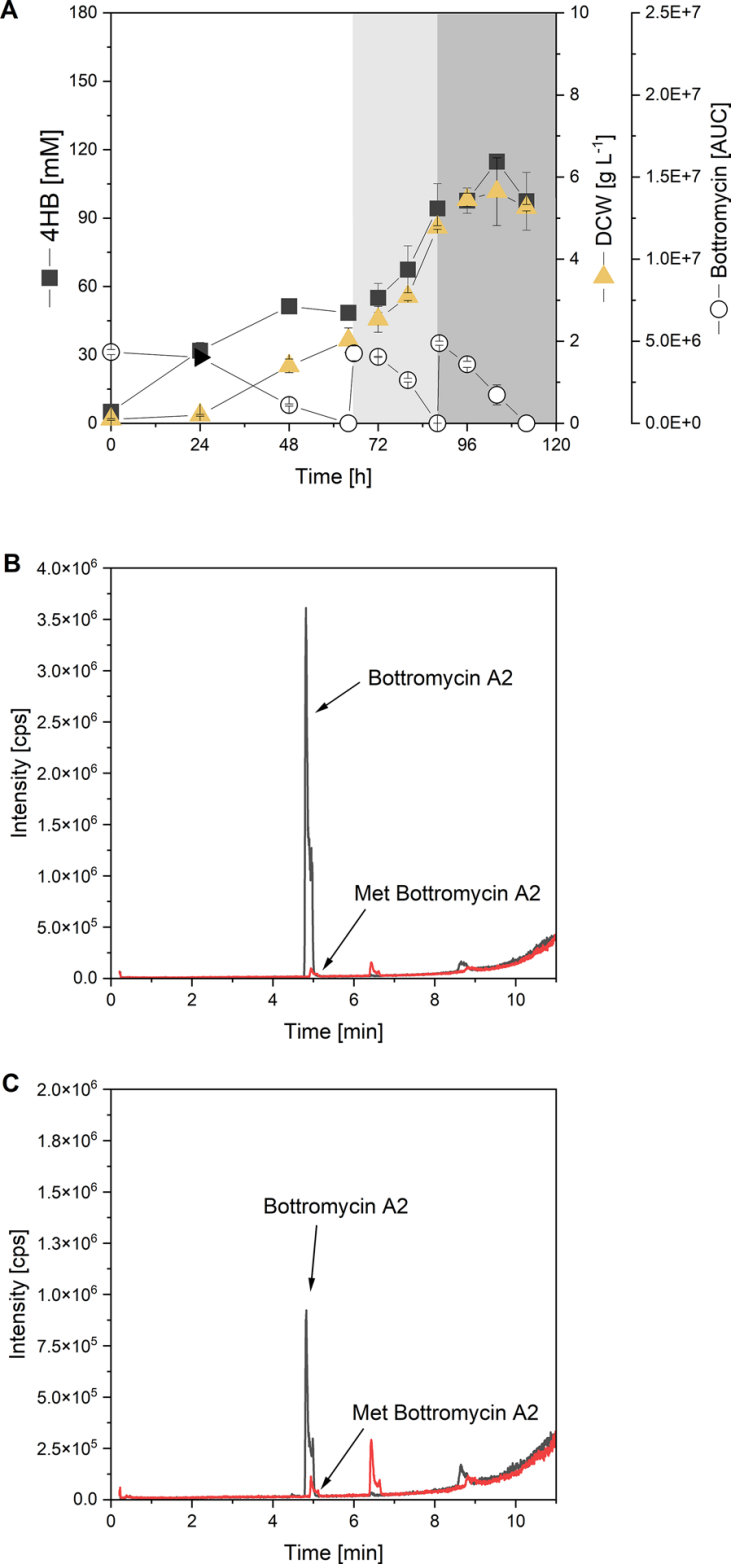
Production of bottromycin A2 from 4-hydroxybenzoate (4HB) in *S. lividans* ΔYA8-DG2 using a fed-batch process with repeated substrate feeding. The data represent the mean values and standard deviations of three biological replicates (n = 3) (**A**). At the end of the process, the product was extracted from the culture broth using ethyl acetate and analysed for the presence of bottromycin A2 [M + H]^+^  = 823.453) and methylated bottromycin A2 [M + H]^+^  = 837.453) using LC–MS (**B**). A commercial bottromycin standard was analysed for comparison (**C**). As shown, a minor fraction of the methylated derivative was formed in addition to bottromycin A2

Furthermore, we explored the production capabilities of *S. lividans* ΔYA8-DG2 grown on protocatechuate (Fig. 3D–F) and 4-hydroxybenzoate (Fig. 3G–I) with starting substrate concentrations up to 30 mM. Remarkably, the strain efficiently processed these toxic aromatic compounds. At 10 mM and 20 mM, protocatechuate was consumed even faster than mannitol. The strain utilized the maximum aromatic level of 30 mM, although this led to initial growth lag and extended cultivation periods on 4-hydroxybenzoate. The resulting biomass was lower than that with mannitol, potentially due to the diminished energy yield from aromatics compared to sugars [40, 41]. Nonetheless, bottromycin A2 was consistently produced across all conditions. This robust production from both aromatic sources and mannitol underscores the promise of *S. lividans* in converting next-generation substrates to bottromycin A2, along with its notable resilience to toxic raw materials. In all cases, production began early and continued steadily regardless of the substrate. This consistent production seemed to be attributed to the synthetic promoter governing bottromycin gene cluster expression, irrespective of the growth phase [20, 21]. Interestingly, the use of 30 mM mannitol resulted in increased synthesis of methylated bottromycin A2 derivatives (24%) as compared to the culture on 30 mM protocatechuate (11%) and 4-hydroxybenzoate (3%). (Additional file 2). Small amounts of methylated bottromycin A2 were also detected in cultures on 10 mM and 20 mM substrate (data not shown).

### Enhanced production of bottromycin A2 from 4-hydroxybenzoate in a fed-batch process

Owing to the high robustness of *S. lividans* ΔYA8-DG2, we tested this heterologous host in a fed-batch process by repeatedly feeding it 4-hydroxybenzoate, the most toxic of the selected substrates. The batch phase was initiated with a concentration of 30 mM 4-hydroxybenzoate, as illustrated in Fig. 4A. We consistently monitored the metabolization of 4-hydroxybenzoate in real time. Upon substrate depletion after 64 h, we reintroduced the compound, raising the concentration back to 30 mM from a concentrated stock. Interestingly, the microorganism consumed this additional amount at a faster rate compared to the initial supply, and it consistently produced bottromycin. It even managed to fully metabolize a third addition of 4-hydroxybenzoate. In the end, the bottromycin A2 levels surged to almost triple of that in the batch process. Low amounts of methylated bottromycin A2 (3% of the amount of the main derivative) was formed in addition (Additional file 2). This showcases the capability of *S. lividans* to upgrade aromatics in a fed-batch process for optimized production. Following this process, we extracted the bottromycin A2 mixture from the culture broth. Analysis of the resultant ethyl acetate extract using LC‒MS/MS revealed that our desired product was identical to a commercial bottromycin standard (Fig. 4B, C).

### The overall metabolism of *S. lividans* exhibited pronounced adaptation in response to different substrates, resulting in varied metabolic gene expression

We subsequently explored the metabolic intricacies of *S. lividans* ΔYA8-DG2 at the systems level by employing transcriptomic studies. For this investigation, cultures were initiated with three different substrates: 10 mM mannitol, protocatechuate, or 4-hydroxybenzoate. We then conducted RNA sequencing. At the 12-h mark, when the cells were sampled for RNA extraction, they demonstrated active growth, carbon consumption, and bottromycin production across all conditions (Fig. 3). The RNA sequencing results showed high statistical quality, as confirmed by PCA and sample distance analysis (Additional file 1: Fig. S2, S3). Notably, each substrate induced a distinct metabolic state at the global gene expression level, with each set of samples clustering separately. With protocatechuate, there was a significant alteration in the expression of 387 genes (which accounts for 5.2% of the total 7497 encoded genes) compared to the mannitol-based culture. Meanwhile, cultures on 4-hydroxybenzoate exhibited changes in the expression of 1786 genes (23.8%) relative to the mannitol culture. Overall, 1373 gene expression variations (18.3%) were observed between the two aromatic substrates (adjusted p value < 0.05, log_2_ fold change ≥ 1), including genes linked to energy and carbon core metabolism (see Additional files 3, 4).

The genes with the most distinct expression variations were closely related to central carbon catabolism (Tables 1, 2). This suggests that the microbe fundamentally altered a critical segment of its metabolism to adapt to the carbon source. Moreover, other genes with pronounced changes were responsible for encoding different metabolic process enzymes, regulators, and some proteins of unspecified function. Specifically, when grown on protocatechuate, the *S. lividans* ΔYA8-DG2 strain showed significant activation of genes associated with TCA cycle enzymes, genes involved in the transformation of phosphorylated intermediates with six and three carbons, belonging to glycolysis and gluconeogenesis, and a series of genes central to aromatic metabolism, including those responsible for the protocatechuate degradation pathway. These include enzymes such as protocatechuate 3,4-dioxygenase, 4-carboxymuconolactone decarboxylase, and 3-carboxy-*cis*, *cis*-muconate cycloisomerase (Table 3). Enzymes of the ethyl-malonyl CoA pathway, a signature pathway among various *Streptomyces* species [42], were also upregulated (Table 4).

**Table 1:** Impact of the growth substrate on global gene expression in *S. lividans* ΔYA8-DG2. The data represent the 40 most strongly upregulated genes during growth on protocatechuate compared to mannitol (p < 0.05, log_2_fold change > 2.0). The encoded enzymes of the TCA cycle (), the EMP pathway, gluconeogenesis (), aromatic catabolism (), and the ethylmalonyl-CoA pathway (). Further genes encode other enzymes (M) and regulators (R), as well as proteins of unknown function (U). The samples were taken from 10 mM cultures after 12 h of incubation (Fig. 2). n = 3

Function	Annotation	Gene	PCA	4HB
R	Two component histidine kinase	SLYA8N_18305	10.2	6.3
	Cytochrome B subunit	SLYA8N_33310	8.7	8.5
M	Glycosyl transferase	SLYA8N_18300	8.1	3.4
M	ABC transporter, ATP-binding protein	SLYA8N_13690	8.0	1.1*
	Succinate dehydrogenase/fumarate reductase iron-sulfur subunit	SLYA8N_33320	7.6	8.5
	Hypothetical protein	SLYA8N_33325	7.4	8.3
M	Protease	SLYA8N_17590	7.3	0.2*
	Succinate dehydrogenase flavoprotein subunit	SLYA8N_33315	7.1	7.6
	Benzaldehyde dehydrogenase	SLYA8N_02130	6.7	8.4
R	Transcriptional regulatory protein	SLYA8N_18310	6.4	2.8
	4-Carboxymuconolactone decarboxylase	SLYA8N_05020	6.4	6.2
	Crotonyl-CoA reductase	SLYA8N_06060	6.3	3.6
	β-Ketoadipyl-CoA thiolase	SLYA8N_05000	6.1	6.2
U	Integral membrane protein	SLYA8N_13695	6.0	0.3*
	Succinyl-CoA:3-ketoacid coenzyme A transferase subunit A	SLYA8N_04990	6.0	6.3
U	Hypothetical protein	SLYA8N_18295	6.0	0.5*
	3-Carboxy-*cis*, *cis*-muconate cycloisomerase	SLYA8N_05015	5.9	5.8
	Succinyl-CoA:3-ketoacid coenzyme A transferase subunit B	SLYA8N_04995	5.9	6.2
	Protocatechuate 3,4-dioxygenase subunit B	SLYA8N_05005	5.8	5.0
	Pyruvate phosphate dikinase	SLYA8N_25215	5.7	6.1
	Transmembrane transporter	SLYA8N_02135	5.7	7.3
	Protocatechuate 3,4-dioxygenase subunit A	SLYA8N_05010	5.7	5.3
R	Response regulator	SLYA8N_17595	5.5	− 0.1*
	Benzoylformate decarboxylase	SLYA8N_02125	5.4	6.6
M	Transport integral membrane protein	SLYA8N_13895	4.8	1.5
	β-Ketoadipyl-CoA thiolase	SLYA8N_04415	4.5	3.8
M	Alcohol dehydrogenase	SLYA8N_29215	4.3	3.8
	Transcriptional regulator	SLYA8N_04985	4.3	4.3
	Protein MeaA	SLYA8N_06065	4.3	3.4
M	Long-chain-fatty-acid-CoA ligase	SLYA8N_04410	4.2	3.6
M	Aldehyde dehydrogenase	SLYA8N_29210	4.0	3.5
M	Exopolysaccharide phosphotransferase	SLYA8N_08030	3.9	2.0
	Oxidoreductase	SLYA8N_06050	3.9	3.3
U	Hypothetical protein	SLYA8N_34285	3.8	3.7
M	Acyl-CoA dehydrogenase	SLYA8N_29235	3.7	3.5
M	Hypothetical protein	SLYA8N_32305	3.6	6.1
M	Iron-sulfur oxidoreductase subunit beta	SLYA8N_05120	3.5	1.9
M	Membrane protein	SLYA8N_03150	3.4	2.4
	Phosphoenolpyruvate carboxykinase	SLYA8N_13500	3.3	3.3
	*p*-Hydroxybenzoate hydroxylase	SLYA8N_22275	3.2	9.0

^*^ Genes not significantly affected (Benjamini‒Hochberg, FDR > 0.05) but included for completeness

**Table 2:** Impact of the growth substrate on global gene expression in *S. lividans* ΔYA8-DG2. The data represent the 40 most strongly unregulated genes during growth on protocatechuate compared to mannitol (p < 0.05, log_2_fold change > 2.0). The encoded enzymes of the EMP pathway are linked to glycolysis and gluconeogenesis (), mannitol catabolism (), and pigment biosynthesis (). Further genes encode other enzymes (M) and regulators (R), as well as proteins of unknown function (U). The samples were taken from 10 mM cultures after 12 h of incubation (Fig. 3). n = 3

Function	Annotation	Gene	PCA	4HB
M	Monooxygenase	SLYA8N_19810	− 5.7	− 0.9*
M	Heavy metal reductase	SLYA8N_19790	− 5.5	− 0.7
M	Arsenite resistance protein ArsB	SLYA8N_19800	− 5.2	− 2.0
R	Transcriptional regulator	SLYA8N_19795	− 5.0	− 2.4
	Substrate binding protein, *smoE*	SLYA8N_28225	− 4.6	− 5.3
	Integral membrane sugar transport protein, *smoF*	SLYA8N_28220	− 4.6	− 5.7
	Hypothetical protein	SLYA8N_28205	− 4.6	− 5.0
	Transcriptional regulator, *smoR*	SLYA8N_28230	− 4.5	− 4.2
	DNA-binding protein	SLYA8N_19785	− 4.4	− 0.6
	Integral membrane sugar transporter, *smoG*	SLYA8N_28215	− 4.4	− 4.3
	Zinc-binding dehydrogenase, *smoD*	SLYA8N_28210	− 4.3	− 3.3
U	Hypothetical protein	SLYA8N_36995	− 4.2	− 5.2
U	Hypothetical protein	SLYA8N_36990	− 4.1	− 5.3
U	Hypothetical protein	SLYA8N_36975	− 4.1	− 5.3
M	Oxidoreductase	SLYA8N_36985	− 4.0	− 5.0
M	Methyltransferase	SLYA8N_36945	− 4.0	− 5.6
M	Endoglucanase	SLYA8N_01130	− 3.9	− 3.0
M	Methylesterase	SLYA8N_36955	− 3.9	− 5.5
	Enolase 2	SLYA8N_01125	− 3.8	− 3.4
	Deoxyribodipyrimidine photo-lyase	SLYA8N_36980	− 3.7	− 5.3
	Phytoene dehydrogenase	SLYA8N_36965	− 3.7	− 4.9
	Geranylgeranyl pyrophosphate synthase	SLYA8N_36970	− 3.7	− 4.2
	Lycopene cyclase	SLYA8N_36940	− 3.7	− 4.7
	Lipoprotein	SLYA8N_36920	− 3.5	− 3.7
	Fructose-specific permease	SLYA8N_21710	− 3.5	− 2.4
M	Secreted protein	SLYA8N_01030	− 3.4	− 0.4*
	DeoR family transcriptional regulator	SLYA8N_21700	− 3.4	− 2.4
	Phytoene synthase	SLYA8N_36960	− 3.3	− 5.2
M	Sigma factor	SLYA8N_36925	− 3.2	− 1.4
	Glyceraldehyde-3-phosphate dehydrogenase	SLYA8N_01755	− 3.2	− 2.6
M	Dehydrogenase	SLYA8N_36950	− 3.2	− 4.8
M	Integral membrane lysyl-tRNA synthetase	SLYA8N_20785	− 3.2	− 1.5
	1-Phosphofructokinase	SLYA8N_21705	− 3.1	− 2.8
U	Hypothetical protein	SLYA8N_36915	− 2.8	− 3.8
M	Secreted protein	SLYA8N_19820	− 2.6	0.1
	Fructokinase	SLYA8N_27935	− 2.6	− 1.8
U	Hypothetical protein	SLYA8N_20790	− 2.5	− 0.9
M	Neutral zinc metalloprotease	SLYA8N_11275	− 2.5	0.3*
M	Lipoprotein	SLYA8N_17715	− 2.4	− 2.9
R	MarR family regulatory protein	SLYA8N_01120	− 2.4	− 2.6

^*^ Genes not significant (Benjamini‒Hochberg FDR > 0.05) but included for completeness

**Table 3:** Impact of the carbon source on the expression of genes encoding substrate uptake and degradation pathways in *S. lividans* ΔYA8-DG2. The strain was grown on minimal medium with 10 mM mannitol, protocatechuate (PCA), or 4-hydroxybenzoate (4HB) as the sole carbon source. Samples were taken from the cultures after 12 h (Fig. 3). The expression levels are normalized to the mannitol-based culture and are given as log_2_-fold change. The significance level (Benjamini‒Hochberg, FDR) was set to < 0.05, n = 3

Gene	Annotation	PCA	4HB
Mannitol uptake			
SLYA8N_09400	Phosphocarrier protein HPr	− 1.48	− 0.87
SLYA8N_17190	Trehalose import ATP-binding protein SugC	− 1.43	− 1.13
SLYA8N_21700	DeoR family transcriptional regulator	− 3.40	− 2.42
SLYA8N_21705	1-Phosphofructokinase	− 3.07	− 2.75
SLYA8N_21710	Fructose-specific permease	− 3.47	− 2.43
SLYA8N_28205	Hypothetical protein	− 4.56	− 5.01
SLYA8N_28210	Zinc-binding dehydrogenase	− 4.28	− 3.31
SLYA8N_28215	Integral membrane sugar transporter	− 4.35	− 4.28
SLYA8N_28220	Integral membrane sugar transport protein	− 4.57	− 5.71
SLYA8N_28225	Substrate binding protein	− 4.61	− 5.28
SLYA8N_28230	Transcriptional regulator deoR-type	− 4.54	− 4.20
Aromatic uptake and degradation			
SLYA8N_02125	Benzoylformate decarboxylase	5.35	6.61
SLYA8N_02130	Benzaldehyde dehydrogenase [NAD( +)]	6.69	8.36
SLYA8N_02135	Transmembrane transporter, Aromatic acid:H^+^ symporter	5.66	7.30
SLYA8N_04990	Succinyl-CoA:3-ketoacid coenzyme A transferase subunit A	6.02	6.29
SLYA8N_04995	Succinyl-CoA:3-ketoacid coenzyme A transferase subunit B	5.89	6.22
SLYA8N_05000	β-Ketoadipyl-CoA thiolase	6.13	6.17
SLYA8N_05005	Protocatechuate 3,4-dioxygenase beta chain	5.78	5.04
SLYA8N_05010	Protocatechuate 3,4-dioxygenase alpha subunit	5.65	5.28
SLYA8N_05015	3-Carboxy-*cis, cis*-muconate cycloisomerase	5.94	5.81
SLYA8N_05020	4-Carboxymuconolactone decarboxylase	6.42	6.19
SLYA8N_22275	*p*-Hydroxybenzoate hydroxylase	3.23	9.01

**Table 4:** Expression levels of genes involved in gluconeogenesis, the phosphoenolpyruvate-pyruvate-oxaloacetate node and the ethylmalonyl-CoA pathway (log_2_ fold change). The strain was grown on minimal medium with 10 mM mannitol, protocatechuate (PCA), or 4-hydroxybenzoate (4HB) as the sole carbon source. Samples were taken from the cultures after 12 h (Fig. 3). The expression levels are normalized to the mannitol-based culture and are given as log_2_-fold change. The significance level (Benjamini‒Hochberg, FDR) was set to < 0.05, n = 3

Gene	Annotation	PCA	4HB
Gluconeogenesis			
SLYA8N_13150	Fructose-1,6-bisphosphatase	2.06	2.98
SLYA8N_13500	Phosphoenolpyruvate carboxykinase [GTP]	3.34	3.28
SLYA8N_35210	Pyruvate carboxylase	1.12	0.46
Phosphoenolpyruvate-pyruvate-oxaloacetate node			
SLYA8N_11390	Pyruvate kinase	− 1.28	− 1.05
SLYA8N_12060	Putative NAD-dependent malic enzyme	1.14	2.06
SLYA8N_22045	Phosphoenolpyruvate carboxylase	− 0.90	− 3.63
SLYA8N_22940	NAD-dependent malic enzyme	0.16*	0.82
SLYA8N_25215	Pyruvate phosphate dikinase	5.70	6.14
SLYA8N_27640	Pyruvate kinase	− 1.03	− 1.57
Ethylmalonyl-CoA pathway			
SLYA8N_04575	Fatty oxidation protein	0.21*	− 0.31*
SLYA8N_04850	Fatty acid oxidative multifunctional enzyme	− 0.52	− 1.04
SLYA8N_04855	Putative acyltransferase	− 0.33*	− 1.27
SLYA8N_06050	Oxidoreductase	3.87	3.31
SLYA8N_06055	Transcriptional regulator	0.41*	− 0.41*
SLYA8N_06060	Crotonyl-CoA reductase	6.28	3.55
SLYA8N_06065	Protein MeaA	4.29	3.42
SLYA8N_06070	Citrate lyase	2.89	2.24
SLYA8N_06075	Hypothetical protein	2.98	2.68
SLYA8N_06080	Acyl-CoA dehydrogenase	1.58	1.15
SLYA8N_06790	Acetyl-/propionyl-coenzyme A carboxylase alpha chain	0.74	− 0.61
SLYA8N_11430	Isobutyryl-CoA mutase A	0.30*	1.42
SLYA8N_11500	Putative acetyl-CoA acetyltransferase	0.65	1.47
SLYA8N_11505	Hypothetical protein	1.03	0.57
SLYA8N_11570	3-Hydroxybutyryl-CoA dehydrogenase	0.58	1.29
SLYA8N_13745	Propionyl-CoA carboxylase beta chain	0.80	− 0.56
SLYA8N_13770	Acetyl-/propionyl-coenzyme A carboxylase alpha chain	0.58	− 0.60
SLYA8N_14030	Methylmalonyl-CoA mutase	0.51	1.30
SLYA8N_14360	Isobutyryl-CoA mutase small subunit	0.42	0.34
SLYA8N_23780	Putative acyl-CoA dehydrogenase	1.24	1.57
SLYA8N_23785	Hydroxymethylglutaryl-CoA lyase	1.09	1.61
SLYA8N_23790	Acetyl-/propionyl-coenzyme A carboxylase alpha chain	0.99	1.26
SLYA8N_23795	Methylcrotonoyl-CoA carboxylase beta chain	1.35	1.51

^*^ Genes not significant (Benjamini‒Hochberg FDR > 0.05) but included for completeness

Conversely, downregulation was noted in a six-gene operon in protocatechuate-fed cells. This operon seems to produce ABC transporter components for mannitol intake based on BLASTN comparison with the genome of the highly related strain *S. coelicolor* A3(2) (Table 5), which corroborated past studies [43]. Additionally, we found reduced expression in genes linked to the fructose phosphotransferase system (PTS). This suggests that mannitol, once it is oxidized in the cytoplasm to fructose, is phosphorylated to fructose 1-phosphate by the fructose PTS. This metabolic mechanism mirrors that in the related actinobacterium *Corynebacterium glutamicum*, as outlined in previous studies [16, 44, 45].

**Table 5:** BLAST analysis of a six-gene operon upregulated during growth of *S. lividans* ΔYA8-DG2 on mannitol against the genome of *S. coelicolor* A3(2). The analysis revealed that the operon encodes a sugar ABC transporter that exhibits similarity to mannitol transporters [43]

Gene	Annotation	Reference	Coverage/Identity (%)
SLYA8N_28205	Hypothetical protein	SCO1902	100/99.0
SLYA8N_28210	Zinc-binding dehydrogenase, *smoD*	SCO1901	100/99.6
SLYA8N_28215	Membrane sugar transporter, *smoG*	SCO1900	100/99.8
SLYA8N_28220	Membrane sugar transport protein, *smoF*	SCO1899	100/100
SLYA8N_28225	Substrate binding protein, *smoE*	SCO1898	100/99.3
SLYA8N_28230	Transcriptional regulator deoR-type, *smoR*	SCO1897	100/99.7

**Fig. 5:**
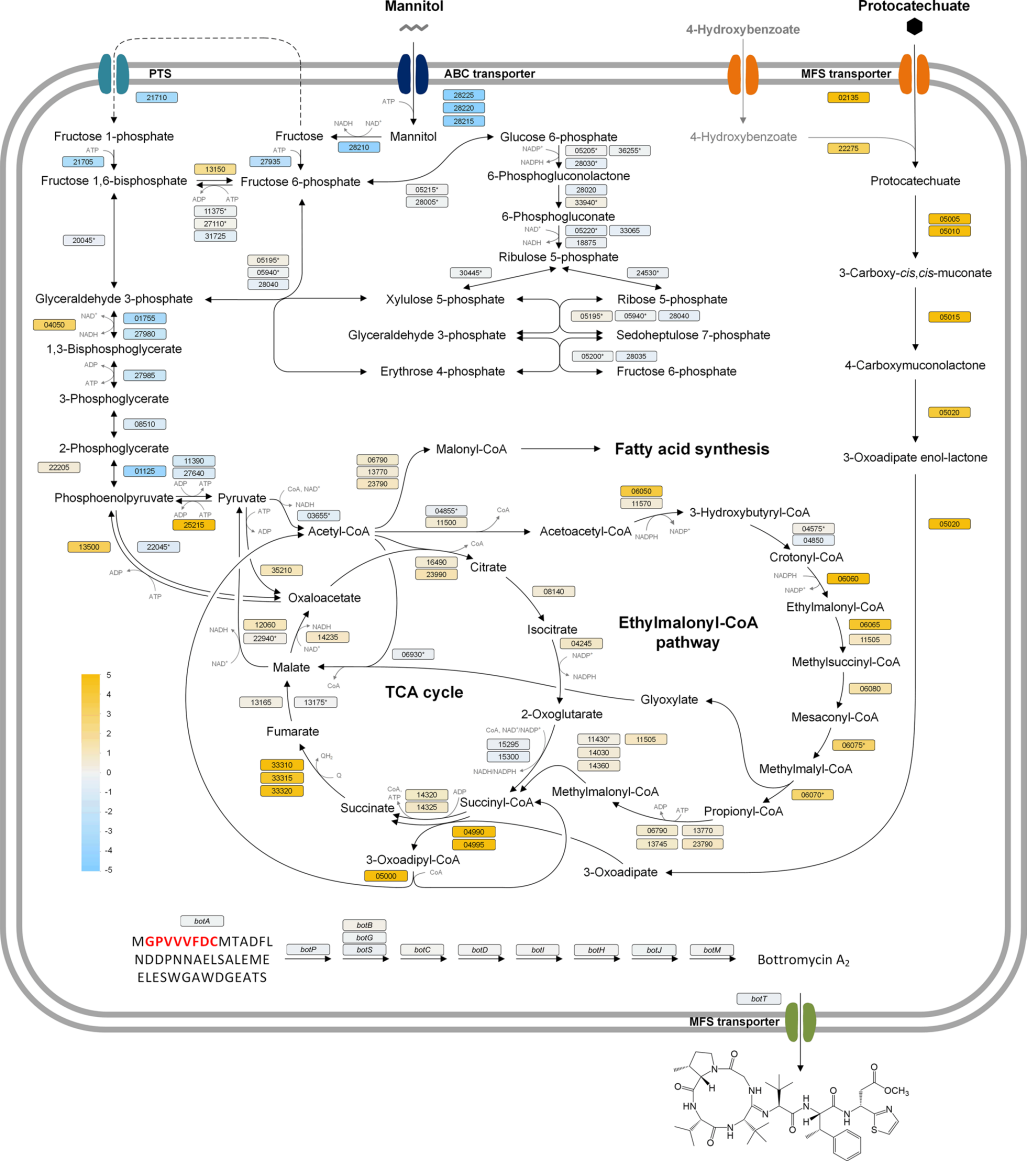
Transcriptome profiling of bottromycin-producing *S. lividans* ΔYA8-DG2 during growth on mannitol and protocatechuate (10 mM). The data display differences in the expression of genes encoding central carbon pathways between mannitol- and protocatechuate-cultured cells after 12 h. The differences are highlighted by colour (blue, downregulated on protocatechuate; yellow, upregulated on protocatechuate). The metabolic network was assembled from the KEGG database, including the following manual curation. The genes encoding the mannitol uptake system were identified in this work (Table 4). Structurally, the uptake system matches that from *C. glutamicum* [16, 103]. The cofactor specificity of phosphoenolpyruvate carboxykinase (PEPCk) and pyruvate carboxylase (PC) was inferred from the corresponding enzymes of *S. coelicolor* M145 [104]. The cofactor specificity of succinate dehydrogenase was taken from *E. coli* [105]. All other cofactors refer to *S. lividans* TK24 [63]. The genes of the ethylmalonyl-CoA pathway were inferred from BLAST analysis (Additional file 1: Table S1). The core peptide of bottromycin is marked in red. ^+^ Putative genes, *Genes not significant (Benjamini‒Hochberg FDR > 0.05) but included for completeness. The data represent mean values and standard deviations from three biological replicates (n = 3)

In terms of metabolic adjustment, the use of mannitol or protocatechuate as the growth substrate corresponded to the activation of their specific uptake systems, deactivating alternative pathways (Fig. 5). This trend was also noted in subsequent reactions related to further substrate metabolism. For cells grown on protocatechuate, a comprehensive catabolic pathway was observed, converting the aromatic substrate to 3-oxoadipyl-CoA and eventually yielding central intermediates such as succinyl-CoA and acetyl-CoA. This influx of succinyl-CoA into the TCA cycle led to the heightened activity of succinate dehydrogenase, pushing carbon towards gluconeogenesis. This is supported by the upregulation of several enzymes, including phosphoenolpyruvate carboxykinase, malic enzyme, and pyruvate phosphate dikinase, among others. In contrast, anaplerotic counterparts such as pyruvate carboxylase were downregulated. The generated acetyl-CoA was channelled into the ethylmalonyl pathway. Notably, when grown on protocatechuate, there was a significant increase in the activity of crotonyl-CoA reductase and ethylmalonyl-CoA mutase, pointing to potential shifts in CoA thioester metabolism (Fig. 5). Finally, for genes associated with amino acid biosynthesis, which is crucial for both general cellular construction and bottromycin formation, gene expression remained largely consistent. This suggests that the cells adeptly managed changes within the core central carbon metabolism (see Additional file 3).

### Efficient bottromycin production from different substrate monomers is driven by stable expression of the biosynthesis-related gene cluster and stable intracellular precursor availability

For genes responsible for bottromycin biosynthesis, no major changes were detected, aligning with the observation that the type of substrate had minimal influence on bottromycin production. In-depth analysis of individual RNA sequence reads demonstrated that the expression patterns within the bottromycin cluster remained consistent regardless of the carbon source employed, as visually depicted in Fig. 2B–D. This underscores the efficacy of the synthetic P_41_ promoter used to guide their stable expression.

**Fig. 6:**
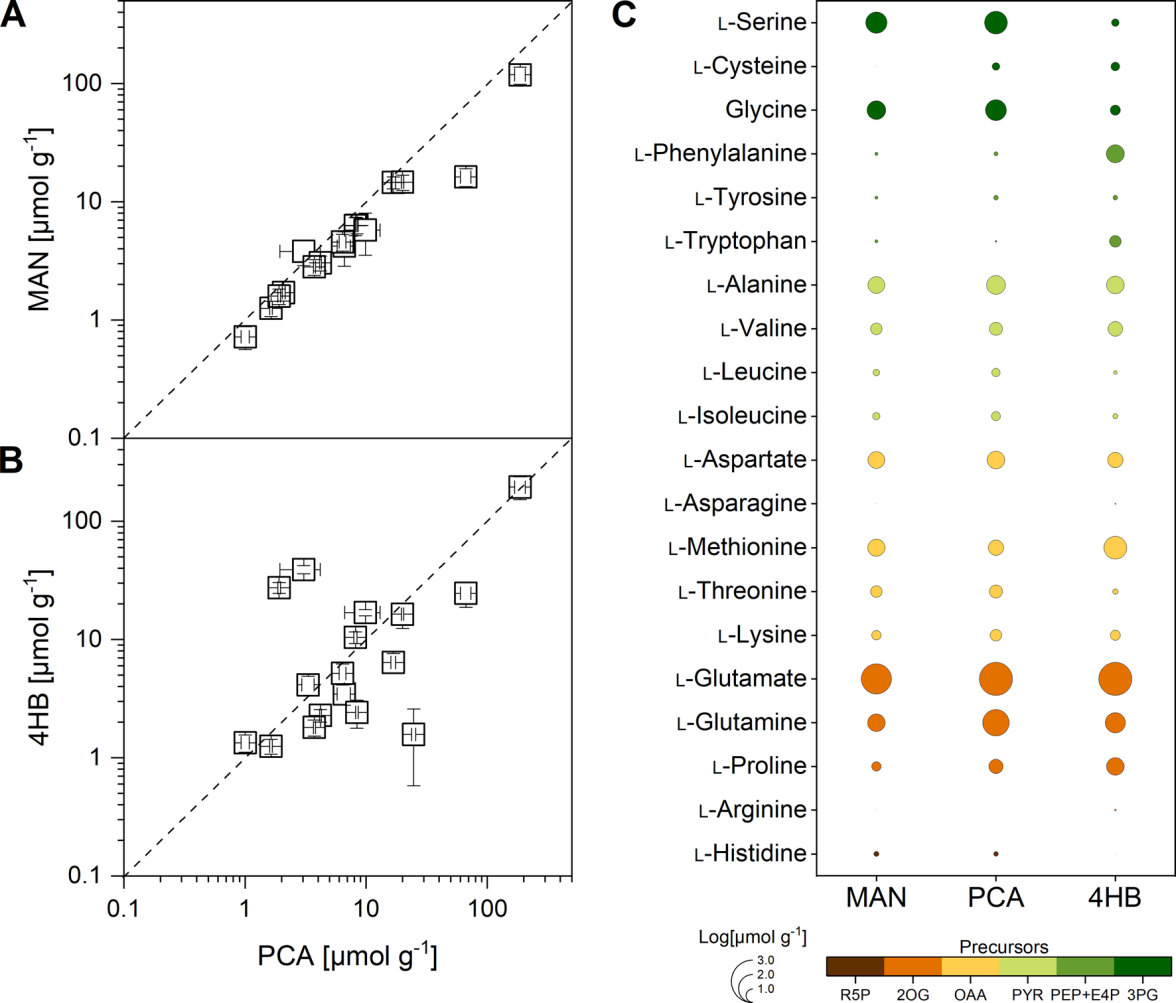
Intracellular amino acid levels of *S. lividans* ΔYA8-DG2 on different substrates. The minimal medium used contained 10 mM mannitol, protocatechuate, or 4-hydroxybenzoate. The data reflect absolute concentrations after 12 h. In addition to the overall comparison between the substrates (**A**, **B**), the data reveal substrate pathway-specific differences related to the biosynthetic origin of the amino acids (**C**). For the latter, the diameter of the circles reflects log-scaled concentrations. The data represent mean values and standard deviations from three biological replicates (n = 3) and analytical duplicates

Our curiosity led us to investigate the strain's behaviour with respect to intracellular amino acid availability. For this purpose, we sampled cultures grown on each of the three substrates after 12 h for metabolomic analysis, aligning with the transcriptomics sampling time point. Intriguingly, the intracellular amino acid pools remained largely unaffected (Fig. 6), with only a few exceptions. Growth on aromatic compounds resulted in slightly elevated levels of l-glutamate, l-glutamine, and l-proline, originating from the 2-oxoglutarate node in the TCA cycle. This response appeared to be correlated with the downregulation of 2-oxoglutarate synthase, ultimately promoting the conversion of this intermediate into the corresponding amino acids. Notably, bottromycin biosynthesis requires the use of 14 distinct amino acids. Clearly, *S. lividans* ΔYA8-DG2 efficiently provides these essential building blocks in a minimal medium, irrespective of whether it is supplemented with mannitol or aromatics.

### The supplementation of aromatic compounds triggers the activation of the ethylmalonyl-CoA pathway in *S. lividans*

As depicted earlier, cells grown on aromatics displayed a notable upregulation of the ethylmalonyl pathway (Fig. 5), a pathway with a wide array of CoA-based intermediates. To gain a deeper understanding of the substrate’s effects and their metabolic repercussions, we opted to quantitatively assess intracellular CoA esters in *S. lividans* ΔYA8-DG2 cultured with 10 mM mannitol, protocatechuate, and 4-hydroxybenzoate. These samples were collected during the early growth and production phase at the 12-h mark.

In addition to the previously investigated CoA-esters [46, 47], our particular interest lies in evaluating the pool of 3-oxoadipyl-CoA, a pivotal intermediate within the aromatic degradation pathway, which has not been assessed before (Fig. 5). Since this metabolite was not commercially available as a reference standard, we explored its enzymatic synthesis. In brief, we cloned and expressed two proteins from *Pseudomonas putida* KT2440 [48], namely**,** 3-oxoadipate CoA-transferase subunit A (PcaI) and 3-oxoadipate CoA-transferase subunit B (PcaJ), in *Escherichia coli*. These efforts yielded a cell extract exhibiting sufficient enzymatic activity to convert the compounds 3-oxoadipate and succinyl-CoA into 3-oxoadipyl-CoA and succinate. The synthesis process yielded 3-oxoadipyl-CoA at 40% of its theoretical maximum yield based on the components used, and this was confirmed through absorption analysis at 260 nm. Following purification, this intermediate was employed to fine-tune the LC‒MS/MS parameters, ensuring high-sensitivity detection. Furthermore, it served as an external standard for the purpose of absolute quantification.

**Fig. 7:**
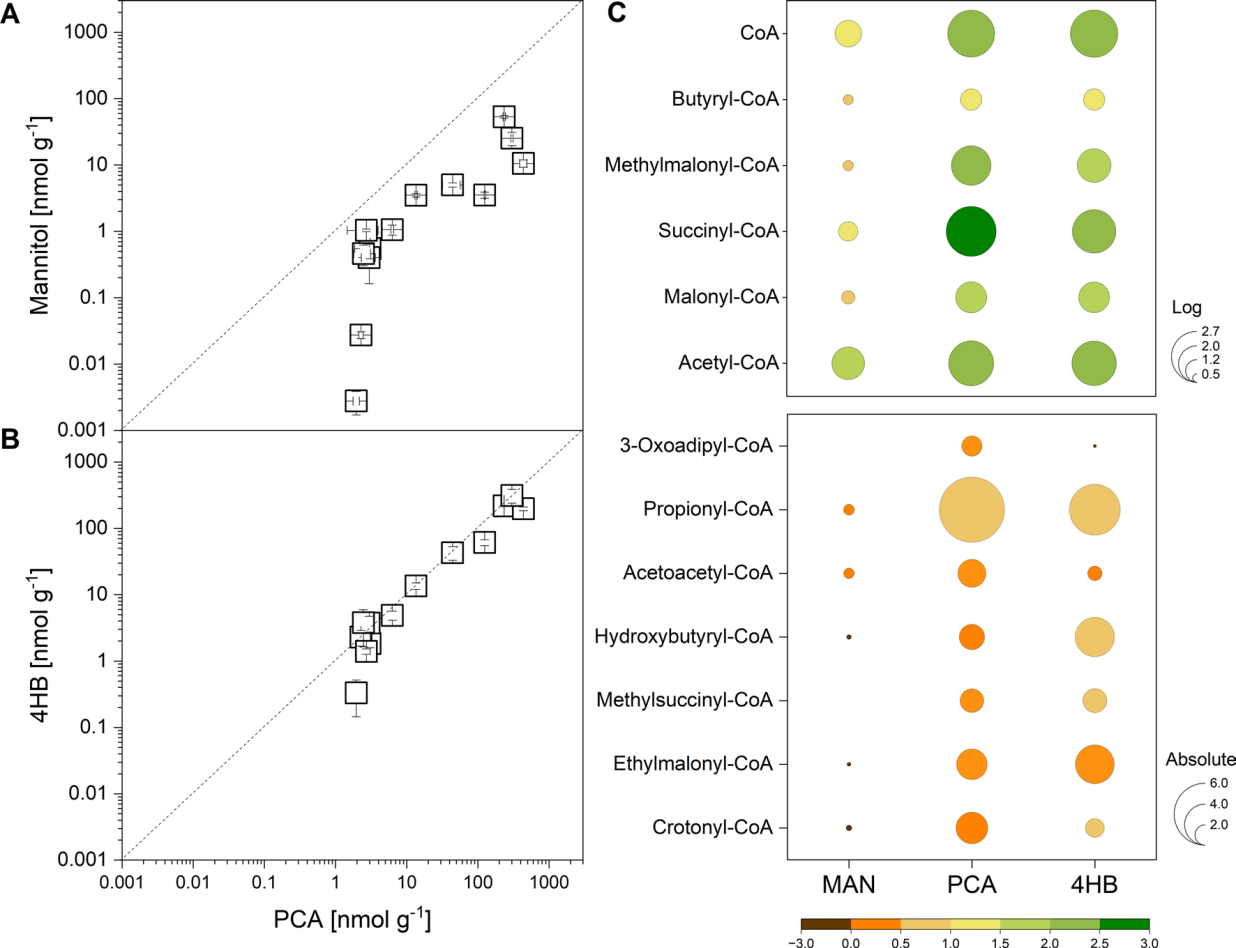
Intracellular CoA-thioester levels of *S. lividans* ΔYA8-DG2. The minimal medium used contained 10 mM mannitol, protocatechuate, or 4-hydroxybenzoate. The data reflect absolute concentrations after 12 h during cell growth on protocatechuate (**A**) and 4-hydroxybenzoate (**B**) compared to mannitol. The size and colour of the circles reflect log-scaled concentrations for high (**C**) and low abundance CoA thioesters (**D**). The data represent mean values and standard deviations from three biological replicates (n = 3) and analytical duplicates

The metabolomics analysis revealed a profound influence of the carbon source on the intracellular composition of CoA esters (Fig. 7). Cells cultivated with mannitol predominantly harboured the "high-abundance" CoA-ester metabolites commonly found in bacteria [46], such as acetyl-CoA, succinyl-CoA, malonyl-CoA, methylmalonyl-CoA, and free CoA, while only minute quantities of other CoA-esters were observed. In stark contrast, cells grown on protocatechuate exhibited substantial deviations. Notably, utilization of the aromatic substrate led to significantly elevated levels of intermediates from the ethylmalonyl-CoA pathway. These included hydroxy-butyryl-CoA, ethylmalonyl-CoA, crotonyl-CoA, butyryl-CoA, methylsuccinyl-CoA, propionyl-CoA, and acetoacetyl-CoA, arranged in order they occur in the pathway. Surprisingly, we detected a notably higher abundance of 3-oxoadipyl-CoA, the first CoA-activated intermediate of protocatechuate degradation. Its pool size was approximately 700-fold higher than that of mannitol-grown cells.

In total, *S. lividans* displayed a CoA-ester pool of 105 nmol g_DCW_^−1^ when cultured with mannitol. However, this pool expanded dramatically to 1173 nmol g_DCW_^−1^, an 11-fold increase, when protocatechuate was employed as the carbon source. An intriguing pattern emerged with the use of 4-hydroxybenzoate. While cells showed a similar trend of the increased abundance of intermediates from the ethylmalonyl-CoA pathway, the presence of 3-oxoadipyl-CoA was significantly lower, approximately sixfold less than in the protocatechuate-grown cells. This disparity might be attributed to differences in the uptake rates of these aromatic compounds. Protocatechuate (10 mM) was depleted at a notably faster rate (Fig. 2D) than 4-hydroxybenzoate (Fig. 2G), potentially leading to a more efficient accumulation of the 3-oxoadipyl-CoA pool. This observation aligns with the slightly lower total CoA-ester pool detected in cells cultivated with 4-hydroxybenzoate, which amounted to 866 nmol g_DCW_^−1^. It should be noted, however, that the 4-hydroxybenzoate-grown cells still contained 119-fold more 3-oxoadipyl-CoA than cells cultured on mannitol.

### The use of aromatic carbon sources impacts the spectrum of pamamycin derivatives in *S. lividans* ΔYA8-R2

Inspired by the observed differences in CoA-thioester abundance (Fig. 7), we decided to investigate the production of polyketides synthetized from these precursors on the different substrates. Pamamycin appeared to be a promising candidate, given its broad spectrum of derivatives that are generated based on the availability of malonyl-CoA, methylmalonyl-CoA, and ethylmalonyl-CoA [39, 49]. The pathway proceeds through 3-oxoadipyl-CoA, a key intermediate in the primary metabolism of the degradation of aromatic compounds [38]. In pamamycin production, the R2 cosmid, housing the pamamycin biosynthesis-related gene cluster under control of its native promoters [38], was integrated into the genome of S*. lividans* ΔYA8 at the *attB* site, bearing the locus tag SLIV19310 (Fig. 2B). This genetic manipulation yielded the engineered strain designated *S. lividans* ΔYA8-R2. Verification of the desired mutation was confirmed through PCR analysis and subsequent sequencing.

**Fig. 8:**
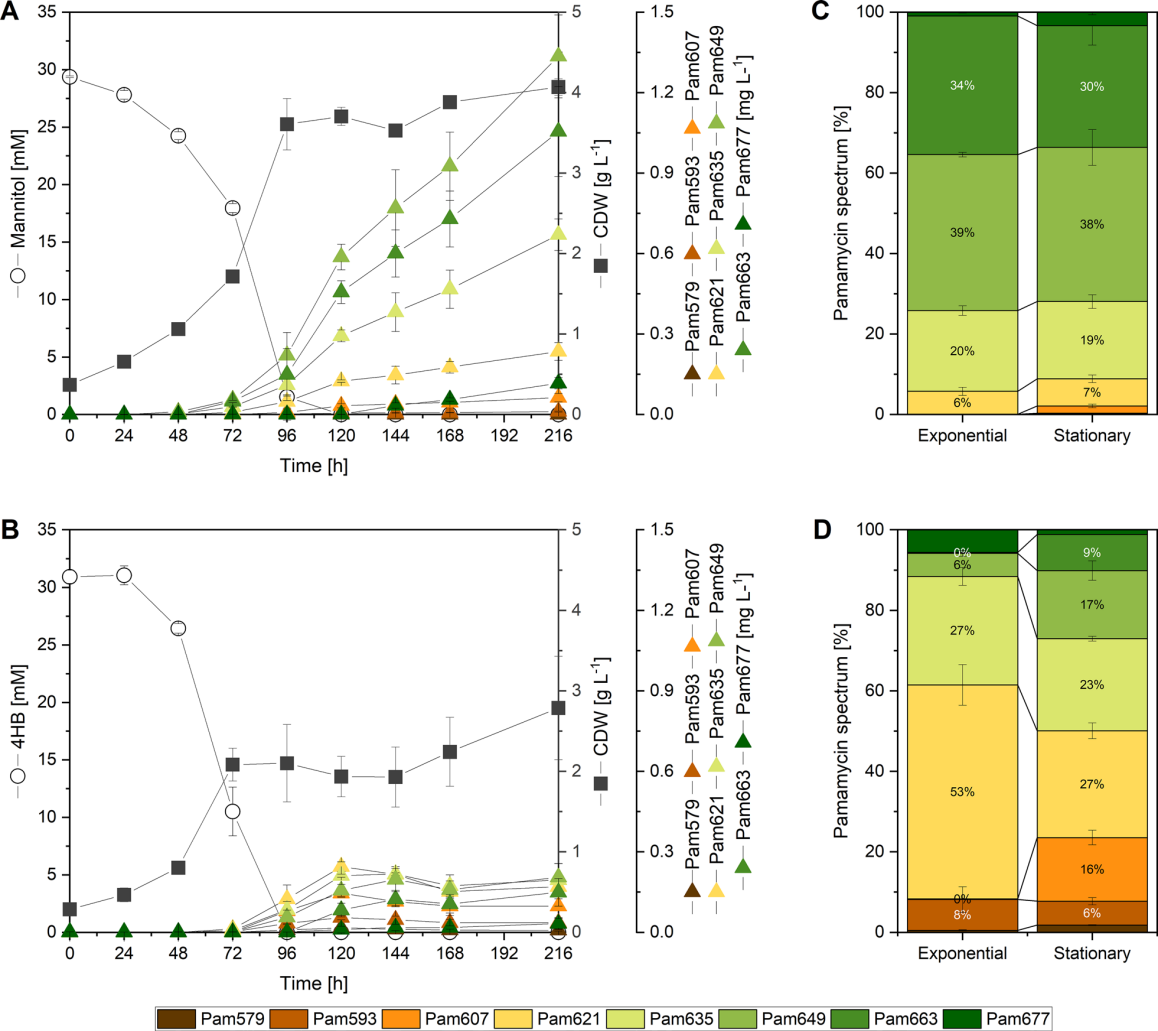
Impact of the carbon source on growth and pamamycin production in *S. lividans* ΔYA8-R2. The recombinant strain was grown in minimal medium containing either 30 mM mannitol (**A**) or 30 mM 4-hydroxybenzoate (4HB) (**B**). In addition, the spectrum of pamamycins formed is given for the mid-exponential (72 h) and stationary phases (216 h) on mannitol (**C**) and 4HB (**D**). The data represent mean values and standard deviations from three biological replicates (n = 3)

This mutant strain exhibited the ability to produce pamamycins when cultivated on two different substrates, namely, mannitol (30 mM) and 4-hydroxybenzoate (30 mM), as evidenced in Fig. 8A and B, respectively. Intriguingly, the production performance displayed notable disparities depending on the carbon sources utilized. On mannitol, the cells exhibited robust growth from an early stage. When grown on 4-hydroxybenzoate, the cells showed an initial lag phase lasting nearly a day due to the compound's inherent toxicity. However, once the cells were past this phase, they rapidly consumed the substrate and accumulated pamamycin. Subsequently, both cultures entered the stationary phase, where cells grown on mannitol continued to accumulate pamamycins, ultimately reaching a total concentration of 3.3 mg L^−1^ after 216 h. In contrast, the 4-hydroxybenzoate cultures did not exhibit further significant production of the natural product, resulting in a final titre of 0.9 mg L^−1^. Notably, the carbon substrate used had a substantial influence on the pamamycin spectrum. On mannitol, the cells generated a substantial fraction of heavy pamamycins, i.e., Pam 649 and Pam 663 (Fig. 8C). Conversely, cultures supplemented with 4-hydroxybenzoate predominantly produced elevated amounts of lighter derivatives such as Pam 621 and Pam 635, accounting for 80% of the total pamamycins (Fig. 8D). Furthermore, during the stationary phase, an intriguing shift in the spectrum of pamamycins was observed.

### Demonstration of pamamycin production from a hydrolysate of the brown seaweed *Himanthalia elongata *using metabolically engineered* S. lividans* ΔYA8-R2

**Fig. 9:**
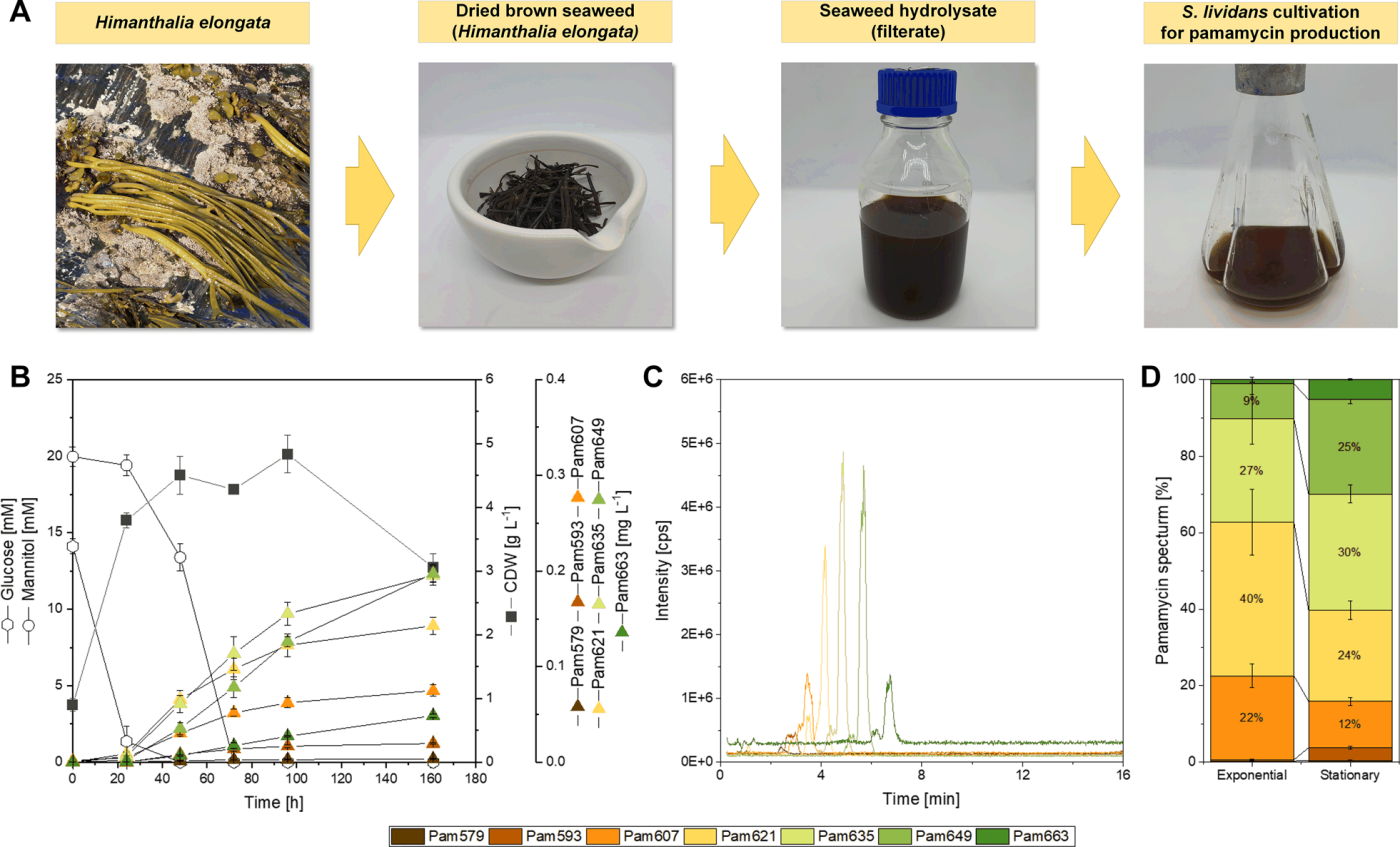
Pamamycin production from a seaweed hydrolysate using *S. lividans* ΔYA8-R2. The hydrolysate was prepared from dried *Himanthalia elongata* [106] (credit for the photo of *H. elongate*: Willem VK, Saxifraga) (**A**). The recombinant strain was cultivated on plain seaweed hydrolysate after pH was adjusted to 7.0 (**B**). Pamamycins in the culture broth were analysed by LC–MS/MS (**C**). In addition, the spectra of pamamycin derivatives during the mid-exponential phase (24 h) and the stationary phase (96 h) are shown. The data represent mean values and standard deviations from three biological replicates (n = 3)

Finally, we aimed to showcase pamamycin production from a seaweed hydrolysate by employing the metabolically engineered strain *S. lividans* ΔYA8-R2. For this purpose, we selected the brown seaweed *Himanthalia elongata*, which exhibits commercial potential, as our primary raw material. The dried seaweed was meticulously processed, including grinding, followed by a gentle enzymatic treatment under slightly acidic conditions (pH 5.5). This process resulted in the generation of an aqueous hydrolysate enriched in mannitol (20 mM) and glucose (14 mM), both of which were byproducts of the digestion process (Fig. 9A). After neutralization with a buffer, this hydrolysate served as our sole source for pamamycin production, without any additional supplementation. Upon inoculating the liquid hydrolysate with strain ΔYA8-R2, we observed a sequential utilization of carbon sources. Initially, glucose was consumed, leading to an increase in biomass concentration to 4 g L^−1^, while total pamamycin production reached 0.02 mg L^−1^ (Fig. 9B). Subsequently, during the mannitol phase, cell growth continued to occur, accompanied by accelerated pamamycin synthesis, ultimately achieving a titre of 0.7 mg L^−1^. Notably, these two distinct culture stages resulted in variations in the pamamycin spectrum. In summary, our endeavour proved successful in demonstrating the feasibility of utilizing seaweed hydrolysate as a viable raw material for pamamycin production, aligning with increased sustainability.

## Discussion

### Streptomyces* lividans* emerges as a robust cell factory for natural products from sustainable resources

The fermentative production of natural products has traditionally been reliant on first-generation carbon sources such as glucose, sucrose, starch, yeast extract, peptone, and soybean meal. In this work, advancements were made beyond this level. As shown, the metabolically engineered *S. lividans* strains successfully produced the antibiotic bottromycin and the insecticide pamamycin from alternative substrates such as 4-hydroxybenzoate, protocatechuate, and mannitol (Figs. 2, 3, 8). Additionally, we showcased compound production from a seaweed hydrolysate rich in mannitol and established the resilience of *S. lividans* during sequential 4-hydroxybenzoate feeding, even though this compound is known to be inhibitory to microbial cells [50]. The three substrates highlighted are gaining traction as sustainable inputs sourced from prominent waste and byproducts. This includes polystyrene waste [14], lignin from biorefineries and the pulp and paper sector [10], and residues from seaweed-based food production and high-value ingredient extraction [11]. For instance, 4-hydroxybenzoate is a significant component in poplars and willows, constituting up to 10% of the lignin fraction [51, 52].

Our findings are a promising step in transitioning microbial natural product production to more eco-friendly raw materials. Economically, there is potential value in this shift. In the industrial sphere, antibiotics can cost as little as 20 US dollars per kilogram. This places them in the specialty chemical category, for which economic viability is heavily influenced by raw material costs. Leveraging affordable byproducts instead of pricier conventional ingredients could enhance production cost-effectiveness. It is worth noting that industrial raw materials, such as those derived from lignin, might bring their own sets of impurities, possibly affecting downstream processing [53]. To achieve even broader use, it seems important to extend the capacity of *S. lividans* to catabolize aromatics. Beyond the two monomers studied here, lignin-derived hydrolysates typically contain a crude mixture of different aromatics such as catechol, phenol, cresols [54], guaiacol [55] and hydroxy-cinnamic acids such as ferulate, caffeate, *p*-coumarate, and vanillate [41]. These compounds cannot be naturally metabolized by *S. lividans,* suggesting the implementation of corresponding routes from other hosts, as shown previously for other bacteria [10].

### The inherent strain metabolic flexibility and the use of a strong synthetic promotor enable stable compound production from different substrates

Previous research on *S. lividans* TK24 has primarily been centred around the synthesis of natural products and enzymes. However, its potential in substrate utilization remains relatively unexplored, and this includes the use of mannitol. For mannitol utilization, studies on the closely related *S. coelicolor* A3(2) strain yielded inconsistent results [43]. While genome analysis of *S. coelicolor* A3(2) hinted at an ABC-type membrane protein designed for sugar alcohols (potentially including mannitol), the corresponding *smo* operon was not triggered by introducing mannitol to a culture medium. A six-gene operon induced in *S. lividans* TK24 during growth on mannitol exhibited high sequence similarity based on BLASTN analysis against the genome of *S. coelicolor* A3(2) (Table 5). Thus, we infer that these genes constitute the mannitol absorption system in both *S. lividans* TK24 and *S. coelicolor* A3(2). Conversely, when protocatechuate and 4-hydroxybenzoate were introduced, the respective catabolic pathways were triggered [10].

Intriguingly, *S. lividans* TK24 cells showcased comparable levels of intracellular amino acids during growth on both an aromatic compound and a sugar alcohol (Fig. 6), even without notable changes in the transcription of amino acid biosynthetic pathways (Additional file 3). This suggests that simply adjusting the high-flux carbon core pathways was sufficient to enable the effective utilization of these distinct substrates. In context, *Bacillus subtilis* primarily modifies its central metabolism to maintain metabolic equilibrium under salt stress [56]. In conclusion, this inherent stability across varied environments, coupled with the adoption of a synthetic promoter for consistent cluster expression (Fig. 2A), seems pivotal for the remarkable production performance observed.

### Aromatic substrates open new avenues for tailor-made product syntheses due to their modulation of the CoA ester spectrum

The metabolism of 4-hydroxybenzoate and protocatechuate resulted in the generation of intermediates of the corresponding protocatechuate 3,4-cleavage pathway, such as 3-oxoadipyl-CoA, succinyl-CoA, and acetyl-CoA. Given the absence of the glyoxylate shunt in *S. lividans* [57]—a typical mechanism for assimilating two-carbon compounds [58]—we theorized that the assimilation of acetyl-CoA might engage the ethylmalonyl-CoA pathway. This hypothesis was supported by the observed upregulation of key enzymes in the ethylmalonyl-CoA pathway: crotonyl-CoA reductase and ethylmalonyl-CoA mutase (Fig. 5). This was further supported by the increase in specific intracellular pathway intermediates in cells grown on aromatics (Fig. 7). The ethylmalonyl-CoA pathway plays a crucial role in the synthesis of polyketides, a commercially significant class of natural products [59], as it supplies the CoA thioester intermediates essential for building these compounds [60].

The pamamycins studied here are notable polyketides known for their intriguing antibiotic activity [36]. They encompass a variety of derivatives marked by structural variations in their side chains at six distinct positions [37, 38]. All pamamycins incorporate succinyl-CoA as a core starter, but their diverse derivatives stem from the alternative addition of three unique CoA thioesters: malonyl-CoA, methyl-malonyl-CoA, and ethyl-malonyl-CoA [38]. Prior research has demonstrated that the pamamycin spectrum in the related strain *S. albus* can be tailored by manipulating the intracellular concentrations of these building blocks. This can be achieved, for example, through genetic alterations of CoA ester metabolism [49] or by feeding the organism branched-chain amino acids [25]. Our findings reveal that the substrate selected significantly influences the intracellular CoA ester profile (Fig. 7). Consequently, this enables the production of diverse pamamycin combinations (Fig. 8). Aromatics tend to drive the synthesis of smaller pamamycin derivatives, whereas mannitol promotes the formation of bulkier versions. This suggests that employing aromatics could be a strategic method to fine-tune the pamamycin spectrum. Undoubtedly, 3-oxoadipyl-CoA played a pivotal role in determining the product spectrum. Cell growth on aromatic compounds led to an accumulation of this uncommon CoA-thioester, which was not observed in cells cultured on mannitol (Fig. 7). In cells grown on aromatics, 3-oxoadipyl-CoA had a twofold role. On the one hand, it emerged during the catabolic breakdown of 4-hydroxybenzoate. On the other hand, it acted as an intermediate in pamamycin biosynthesis, stemming from the union of malonyl-CoA and succinyl-CoA. This particular interaction was instrumental in producing lighter pamamycin derivatives [38]. Given the swifter metabolism of protocatechuate compared to 4-hydroxybenzoate and the substantial increase in the 3-oxoadipyl-CoA pool (Fig. 7), it is plausible that using protocatechuate as a substrate could further amplify the selective synthesis of lighter pamamycins. For a more comprehensive understanding, in future studies, the enzymatic synthesis of methyl-3-oxoadipyl-CoA should be explored, paralleling the approach taken for oxoadipyl-CoA in our study, to facilitate its LC‒MS analysis in cell extracts. This derivative plays a significant role in the pamamycin pathways, guiding the formation of heavier product variants (Fig. 1). In addition, the substrates had an influence on the bottromycin spectrum (Additional file 2).

Beyond pamamycins, *S. lividans* [22] is a well-known heterologous host to produce natural products and proteins, which is attributed to its acceptance of methylated DNA, its low endogenous protease activity [17, 61], the availability of *clean* strains that have been cleared of their native natural product gene clusters [18], and the development of promoters for tuneable promotion [62]. This positions *S. lividans* as an optimized host strain for heterologous expression. In this regard, it seems promising to evaluate *S. lividans* to produce other compounds from, what one might call, *sustainable* monomers. As an example, a stable supply of amino acids in the different substrates (Fig. 6) seems to be a valuable trait to synthetize heterologous proteins such as cellulase [63] and human proteins [64] in the host.

### The synthesis of natural products from sustainable raw materials promises to be an attractive complement to existing biorefineries

Integrating the production of high-value bioactive natural products into existing biorefineries, which have traditionally focused on low-value bulk chemicals, would offer several compelling advantages. By producing both high- and low-value products, biorefineries can diversify their portfolio, enhancing economic stability in a still challenging market [65, 66]. With regard to the valorisation of lignin, the previously demonstrated production of bulk chemicals such as *cis*, *cis*-muconic acid [41, 67, 68], β-ketoadipic acid [69, 70], lactate and pyruvate [71], and aromatic monomers such as ferulic acid and *p*-coumaric acid as antioxidants [72] could be well complemented by high-value natural products. For seaweed biorefineries, natural product synthesis from algal residuals could be linked into cascaded value chains that sequentially extract high-value ingredients and then ferment the resulting hydrolysates and extracts into value-added chemicals [11]. Thus far, the latter addresses biofuels and bulk chemicals such as ethanol [28, 73, 74], butanol [75, 76], lactate [77], and L-lysine [16], offering promising opportunities for additional value creation by natural product synthesis. It is worth mentioning that when demonstrating pamamycin production in our work, we mimicked the sugar- and mineral-rich residual streams that typically accumulate as a side product from the extraction of algal biomass [78]. This increased diversification and economic resilience would help to buffer biorefineries against market fluctuations in the bulk chemical sector and add revenue streams from premium products [79].

## Conclusions

High-value natural products, especially those with therapeutic, cosmetic, or specialized industrial applications, have a growing demand. Notably, the global spending on pharmaceuticals expanded by 56% from 2007 to 2017 [80]. By meeting this demand and integrating microbial production of these types of products, biorefineries could place themselves in advantageous position in the market. In this regard, our findings provide an exciting proof-of-principle for expanding this field. However, one should note that the process is far from being industrially implementable yet. Significant research and development efforts are needed to meet the challenges ahead [81, 82]. On the technological side, these factors relate to reducing energy costs during raw material pretreatment, e.g., drying seaweed [11] or efficiently depolymerizing the rigid structure of lignin [83]. With respect to scaling, the obtained hydrolysates are rather dilute and contain only low levels of available carbon-limiting product titres and demand high-volume fermentation equipment [84]. Furthermore, in practice, raw materials from waste and residual streams are crude mixtures of substrates and might contain toxins, exceeding the capabilities of the cell factories used [85]. In addition, the supply of lignin and seaweed used in industries, except for lignin that is derived from pulp and paper plants, are not centralized, and require the implementation of new structures [86]. To address these issues associated with industrial implementation, we need new interdisciplinary value chains [16, 41, 54, 67]. In this regard, a shift towards producing complex natural products from new raw material streams seems promising to drive innovation, potentially leading to the discovery of new processes, products, or applications and opening doors for collaborative research and development [79].

In addition to *S. lividans*, other *Streptomyces* and related strains that are potent in forming natural products should also be considered in future studies. *Streptomyces* are known to grow well on mannitol and glucose, which are found in seaweed hydrolysates [87]. Furthermore, they contain pathways to degrade a range of other sugars potentially contained in algal streams, such as mannose, galactose, and rhamnose [11], suggesting that seaweed biomass could be valorised in a rather straightforward manner. On the other hand, the spectrum of metabolized aromatics differs quite substantially between strains. As an example, *Amycolatopsis* sp. can degrade benzoate, phenol, and guaiacol [55], while *S. albus* lacks the β-ketoadipate pathway necessary for aromatics utilization [88]. In this regard, metabolic engineering strategies for the conversion of aromatic mixtures and aromatic-rich waste streams is likely to be rather strain specific.

## Material and methods

### Microorganisms and plasmids

*Streptomyces lividans* ΔYA8 was obtained from previous work [18]. DNA of *Pseudomonas putida* KT2440 was utilized to amplify the two genes *pcaI* and *pcaJ*, encoding 3-oxoadipate:succinyl-CoA transferase [48]*. Escherichia coli* DH5α (Invitrogen, Carlsbad, USA) was employed for plasmid amplification. *E. coli* ET12567/pUZ8002 was used for conjugal gene transfer [23]. *E. coli* BL21 (DE3) (Thermo Fisher Scientific, Karlsruhe, Germany) and the plasmid pET-21a (Invitrogen) were used for protein expression. The cosmids DG2-km-P41hyg (bottromycin biosynthesis-related gene cluster under synthetic promoter control) [20] and R2 (pamamycin biosynthesis-related gene cluster under native promoter control) [38] were taken from previous work. All strains were stored in 20% glycerol at −80 °C. All strains and plasmids are listed in Table 6.

**Table 6:** Strains and plasmids

Strains and plasmids	Description	References
Strains		
*E. coli* DH5α	Strain for plasmid amplification	Invitrogen
*E. coli* ET12567	Strain harbouring pUZ8002 for conjugal gene transfer	[23]
*E. coli* BL21 (DE3)	Strain for high-level expression of recombinant proteins	Thermo Fisher Scientific
*E. coli* p21pcaI	Derivative of *E. coli* BL21 (DE3) harbouring p21pcaI gene	This work
*E. coli* p21pcaJ	Derivative of *E. coli* BL21 (DE3) harbouring p21pcaJ gene	This work
*S. lividans* ΔYA8	Derivative of *S. lividans* TK24, 8 secondary metabolite gene clusters were removed from genomic DNA	[18]
*S. lividans* ΔYA8-DG2	Derivative of *S. lividans* ΔYA8 containing bottromycin biosynthetic cluster	This work
*S. lividans* ΔYA8-R2	Derivative of *S. lividans* ΔYA8 containing pamamycin biosynthetic cluster	This work
Plasmids		
pET21a	Vector for protein overexpression	Invitrogen
p21pcaI	Derivative of pET21a plasmid. Protein expression vector for pcaI gene of *P. putida* KT2440	This work
p21pcaJ	Derivative of pET21a plasmid. Protein expression vector for pcaJ gene of *P. putida* KT2440	This work
DG2-Km-P41hyg	Derivative of integrative DG2-cosmid with Km^R^ marker and P41 promoter pairs, contains bottromycin biosynthetic cluster	[20]
R2	An Integrative cosmid containing pamamycin biosynthetic gene cluster	[38]

### Media

LB medium (Becton & Dickinson, Heidelberg, Germany) and terrific broth were used to culture *E. coli*. The terrific broth contained 24 g of yeast extract (Sigma‒Aldrich, Taufkirchen, Germany), 12 g of tryptone (Fluka, Buchs, Switzerland), 5 g of glycerol, 12.5 g of K_2_HPO_4_, and 2.3 g of KH_2_PO_4_ per litre. Plate cultures of *S. lividans* were grown on mannitol soy (MS) flour agar containing 20 g of mannitol, 20 g of soy flour (Schoenenberger Hensel, Magstadt, Germany), and 20 g of agar (Becton & Dickinson) per litre. Liquid cultures of *S. lividans* involved two sequential precultures, followed by the main culture. Tryptic soy broth (30 g L^−1^, TSB, Sigma‒Aldrich) was used for the first precultivation. The second precultivation and the main cultivation were conducted in minimal medium [46] containing 200 mM potassium phosphate buffer (pH 7.8): 15 g of (NH_4_)_2_SO_4_, 1 g of NaCl, 200 mg of MgSO_4_·7H_2_O, 55 mg of CaCl_2_, 20 mg of FeSO_4_·7H_2_O, 2 mg of FeCl_3_·6H_2_O, 2 mg of MnSO_4_·H_2_O, 0.5 mg of ZnSO_4_·H_2_O, 0.2 mg of CuCl_2_·2H_2_O, 0.2 mg of Na_2_B_4_O_7_·10H_2_O, 0.1 mg of (NH_4_)_6_Mo_7_O_24_·4H_2_O, 1 mg of riboflavin, 1 mg of nicotinamide, 0.5 mg of thiamine hydrochloride, 0.5 mg of pyridoxine hydrochloride, 0.2 mg of biotin, and 0.1 mg of *p*-aminobenzoate per litre. In parallel, mannitol, protocatechuate (PCA), and 4-hydroxybenzoate (4HB) were added as the sole carbon sources, as given below. When needed, kanamycin (50 μg mL^−1^), apramycin (20 μg mL^−1^), phosphomycin (200 μg mL^−1^), and ampicillin (100 μg mL^−1^) were added to filter sterilized stocks.

### Genetic engineering

SnapGene software (GSL Biotech LLC, San Diego, USA) was used for strain and primer design. Transformation and conjugation of S. *lividans* ΔYA8 was based on standard methods [23]. The site-specific integration of biosynthesis-related gene clusters into the chromosome of S. *lividans* ΔYA8 was carried out using the phiC31 integrase system [20] and site specific primers (Additional file 1: Table S2). In short, the corresponding cosmid was transformed into *E. coli* DH5α using heat shock, amplified, isolated (QIAprep Spin MiniPrep Kit, Qiagen, Hilden, Germany), and transformed into *E. coli* ET12567/pUZ8002 by electroporation. The obtained mutant then served as a donor for conjugal transfer. For this purpose, it was mixed with spores of *S. lividans* ΔYA8, plated on MS agar, and incubated at 30 °C overnight. For the selection of transconjugants, the agar was then overlaid with phosphomycin and selective antibiotics. Afterwards, the plates were further incubated until sporulation. The obtained transformants were verified for correctness of the desired genetic change by PCR (Phire Green Hot Start II PCR Mastermix, Thermo Scientific, Waltham, MA, USA) and by sequencing. Furthermore, we created *E. coli* mutants to enable the enzymatic synthesis of 3-oxoadipyl-CoA, which is needed as a standard for its intracellular analysis by LC‒MS/MS [46]. For this purpose, we selected the genes *pcaI* and *pcaJ* encoding 3-oxoadipate:succinyl-CoA transferase in the genome of *P. putida* KT 2440 [48]. First, the backbone of the pET-21a expression vector was digested with FastDigest NdeI and HindIII (Thermo Fisher Scientific) according to the manufacturer’s protocol. Then, the two genes of *P. putida* KT 2440 were separately amplified from genomic DNA (Q5 HotStart Polymerase, New England Biolabs, Frankfurt am Main, Germany). Following amplification, each gene was cloned and inserted into the vector (Gibson Assembly MasterMix, New England Biolabs). Vector overhangs are underscored. Subsequently, the plasmids were transformed into *E. coli* DH5α, amplified, isolated, and verified by sequencing. *E. coli* BL21 (DE3) was then transformed with the expression plasmids using heat shock.

### Preparation of brown seaweed hydrolysate

To prepare mannitol-rich seaweed hydrolysate, 50 g of dried *Himanthalia elongata* (PureRaw, Klötze, Germany) was blended into powder, suspended in 500 mL of deionized water, and extracted (121 °C, 18 min) [16]. Celluclast 1.5 L and Viscozyme L (Sigma‒Aldrich, Steinheim, Germany) were added to the mixture at 0.01 g of the enzyme mix per g of dry biomass, followed by pH adjustment to 5.5. The mixture was incubated over 48 h. Afterwards, the hydrolysate was clarified (4500 ×*g*, 15 min, 4 °C), and the solution pH was adjusted to pH 7.0 (6 M NaOH). The obtained solution was autoclaved prior to further use.

### Batch cultivation in shake flasks

Cultivations were conducted in 500 mL baffled shake flasks filled with 50 mL medium and 30 g soda-lime glass beads (5 mm, Sigma‒Aldrich) on an orbital shaker (230 rpm, 28 °C, 75% relative humidity, 5 cm shaking diameter, Multitron, Infors AG, Bottmingen, Switzerland). For bottromycin production, 10^7^ spores of the corresponding producer were inoculated into TSB medium and incubated for 48 h. Then, the cells were harvested (8000 ×*g*, 2 min, 25 °C) and transferred to the second preculture in minimal medium containing 10 g L^−1^ mannitol as the carbon source, followed by incubation over 72 h, harvesting, and inoculation of the main culture, which contained either mannitol, PCA or HBA, as specified below. For pamamycin production, 10^7^ spores of the corresponding producer were inoculated into TSB medium and incubated for 24 h. Then, either 10 mM mannitol or 4-HB was added to the preculture for adaptation, and the preculture was further incubated for an additional 24 h. Cells were collected (8000 ×*g*, 2 min, 25 °C) and inoculated into the main culture in minimal medium containing mannitol or 4HB. Generally, the main cultures were inoculated to a starting optical density (OD_600_) = 0.5. All cultivations were carried out in triplicate.

### Batch cultivation on seaweed hydrolysate

First, 90% (v/v) seaweed hydrolysate was mixed with 10% (v/v) 2 M MOPS buffer (pH 7.0). As described above, 10^7^ spores of the pamamycin producer were inoculated into TSB medium and incubated for 48 h. Then, the cells were centrifuged (8000 ×*g*, 2 min, 25 °C) and inoculated into seaweed hydrolysate medium.

### Fed-batch cultivation in shake flasks

First, 10^7^ spores of the bottromycin-producing mutant were inoculated into TSB medium and incubated for 48 h. Then, the cells were collected (8000 ×*g*, 2 min, 25 °C) and inoculated into minimal medium containing 10 g L^−1^ mannitol. The second preculture was grown for 72 h and then used to inoculate the main culture containing 30 mM 4HB. When the substrate was completely consumed, a pulse of the substrate was added from a concentrated stock (500 mM, pH 7.0), which increased the 4HB level in the broth to 30 mM.

### Quantification of cell concentration

Generally, growth was inferred from optical density (OD) based on photometric measurements at 600 nm. In addition, the dry cell weight (CDW) was measured gravimetrically. For this purpose, cells were harvested (10,000 ×*g*, 4 °C, 10 min), washed with 15 mL deionized water, freeze-dried, and weighed [39]. Systematic measurements provided a substrate-specific correlation between OD_600_ and CDW, which allowed us to infer the latter from OD readings: CDW (g L^−1^) = 0.946 × OD_600_ (mannitol, 4HB), CDW (g L^−1^) = 0.474 × OD_600_ (PCA).

### Quantification of substrates

Sugars were analysed by HPLC (1260 Infinity Series, Agilent, Waldbronn, Germany) using a column (NUCLEOGEL SUGAR Pb, 300 × 7.8 mm, Macherey–Nagel, Düren, Germany) at 80 °C as the stationary phase and deionized water as the mobile phase (0.4 mL min^−1^). Refraction index measurements and external standards were used for quantification. HPLC-based analysis of aromatics (PCA, 4HB) involved separation on a C18 column at 25 °C (Nucleodur C18 Isis, 100 × 3 mm, Macherey–Nagel) with a gradient of 0.025% H_3_PO_4_ and acetonitrile (1 mL min^−1^) [55]. The analytes were detected by UV absorbance at compound-specific wavelengths (210 nm for PCA, 260 nm for 4HB). External standards were used for quantification.

### Quantification of intracellular amino acids

Intracellular amino acids were quantified as previously described [89, 90]. In brief, 2 mg of biomass was harvested and vacuum-filtered (cellulose nitrate, 0.2 μm pore size, 47 mm, Sartorius, Göttingen, Germany). The filter with the cells was washed (15 mL 2.5% NaCl, 25 °C) and quickly transferred into 2 mL of a 200 μM α-aminobutyrate solution, followed by extraction in boiling water (15 min, 100 °C). Afterwards, the extract was cooled on ice and clarified from debris (20,000 ×*g*, 5 min, 4 °C). The supernatant was used for analysis. For this purpose, the amino acids were separated by HPLC on a reversed-phase column (Gemini 5 µm C18 110 Å, 150 × 4.6 mm, Phenomenex) after precolumn derivatization with *o*-phthaldialdehyde and fluorenylmethyloxycarbonyl chloride [91]. Quantification was based on *α*-aminobutyrate as an internal standard.

### Analysis of bottromycin and pamamycin

In brief, 300 μL of culture broth was mixed with 300 μL of acetone and shaken for 10 min at room temperature (1000 rpm, Thermomixer F1.5; Eppendorf, Wesseling, Germany). Then, 300 μL of ethyl acetate was added, and the mixture was incubated under the same conditions for another 10 min. The organic phase was separated (20,000 ×*g*, room temperature, 10 min), collected, and evaporated under nitrogen. The obtained extract was dissolved in 300 μL methanol, clarified by centrifugation (20,000 ×*g*, 4 °C, 10 min) and analysed by LC–ESI–MS (Agilent Infinity 1290; AB Sciex QTrap 6500, Darmstadt, Germany) [20, 21, 46]. Separation of the analytes was conducted using a reversed-phase column (Vision HT C18 HighLoad, 100 × 2 mm, Dr. Maisch, Ammerbuch-Entringen, Germany) operated at 45 °C at a flow rate of 0.55 mL min^−1^ with the following linear gradient of 0.1% formic acid in deionized water (A) and 0.1% formic acid in acetonitrile (B): 0–11 min, 95–5% A, 5–95% B. Based on LC–ESI–MS analysis [20], bottromycin A2 ([M + H] +  = 823.453) was the major derivative formed, while methylated bottromycin A2 [M + H] +  = 837.453) accumulated in traces. As a reference, a commercial standard (purity 85%) was obtained from Cayman Chemicals (Ann Arbor, MI, USA). Pamamycins were separated using 8 mM ammonium formate in 92% acetonitrile at a flow rate of 0.3 mL min^−1^ and analysed by LC–ESI–MS as described previously [39]. The chromatographic and mass spectrometric settings for natural product analysis are described in Additional file 1: Table S3.

### Enzymatic synthesis and purification of 3-oxoadipyl-CoA

*Escherichia* *coli* BL21 (DE3) harbouring p21pcaI and p21pcaJ, respectively, was plated on LB agar supplemented with the appropriate antibiotics. After overnight incubation at 37 °C, a single colony of each mutant was inoculated in 10 mL of antibiotic-amended LB medium (100 mL shake flask), grown overnight on an orbital shaker (37 °C, 160 rpm) and inoculated into 1 L of terrific broth containing the respective antibiotics (3 L shake flask). When the OD reached 0.7, the cultures were cooled to 23 °C, and protein production was induced by the addition of IPTG to a level of 0.5 mM. The incubation was continued overnight. Afterwards, the cells were harvested (5000 ×*g*, 4 °C, 45 min) and resuspended in buffer A (50 mM HEPES–KOH, 450 mM NaCl, 15% (v/v) glycerol, pH 7.6) at a ratio of 2:1 (v/w), followed by sonication for cell lysis. Cell debris was removed by centrifugation (55,000 ×*g*, 4 °C, 45 min). The supernatant was purified using Ni–NTA agarose beads (Protino, Macherey–Nagel, Düren, Germany), dialyzed over a PD-10 column (Cytiva, Freiburg, Germany), and concentrated (Amicon centrifugal filter, Merck, Darmstadt, Germany), and the protein size was verified via SDS‒PAGE (PcaI = 26.6 kDa; PcaJ = 24.7 kDa). The protein concentration was determined spectroscopically at 260 nm using the corresponding molar extinction coefficient [92]. All CoA-thioesters were purified by a 1260 Infinity LC system (Agilent) using a reversed-phase column (Gemini 10 μm NX-C18 110 Å, 100 × 21.2 mm AXIA packed column, Phenomenex, Aschaffenburg, Germany). Succinyl-CoA was obtained using previously established methods [93], wasy purified by a gradient of solvent A (25 mM NH_4_HCO_2_, pH 4.2) and solvent B (methanol) at a flow rate of 25 mL min^−1^: 5–23% B in 15 min, followed by 3 min washing (95% B) and subsequent re-equilibration of the column for 3 min (5% B), and was then used as a substrate. For enzymatic synthesis of 3-oxoadipyl-CoA, 30 mg of succinyl-CoA (8.25 mM final concentration, 1 eq.) was dissolved in 25 mM ammonium formate (pH 4.2) and added to a mixture containing 33 mM 3-oxoadipic acid (4 eq.), 20 mM MgCl_2_, 100 mM KHCO_3_ and 100 mM HEPES–KOH (pH 7.5) at a final volume of 4 mL. After brief equilibration at 30 °C, 10 µM of each PcaI and PcaJ was added, and the mixture was further incubated (30 °C, 200 rpm). After 3 h, the assay was quenched with a final concentration of 10% (v/v) formic acid, centrifuged (4500 × g, room temperature, 20 min), and filtered (0.45 µm). The purification of 3-oxoadipyl-CoA was conducted by using solvent A (50 mM NH_4_CH_3_CO_2_, pH 8.1) and solvent B (methanol) at isocratic flow (2.5% of solvent B) for 17 min with a flow rate of 25 mL min^−1^, followed by 3 min washing at 95% B and 3 min re-equilibration with 2.5% B. Fractions containing either product were pooled, flash-frozen in liquid nitrogen, lyophilized and stored at − 20 °C. The obtained concentrations were determined photometrically, considering the known extinction coefficient of saturated acyl-CoA (ε_260 nm_ = 16.4 mM^−1^ cm^−1^) [93, 94].

### Analysis of intracellular CoA thioesters

Intracellular CoA thioesters were quantified using a recently established protocol [39, 46] with slight adaptations. In short, cell broth, containing approximately 8 mg CDW, was transferred into a quenching solution (95% acetonitrile with 25 mM formic acid, − 20 °C) at a volume ratio of 1:2, mixed and kept on ice for 10 min. Cell debris was removed (10 min, 4 °C, 10,000 ×*g*). The supernatant was transferred into 5 ml of supercooled deionized water. The remaining pellet was washed once with supercooled deionized water. Then, the two supernatants were combined, frozen in liquid nitrogen, and lyophilized. The obtained extract was resuspended in 1 mL of resuspension buffer (25 mM ammonium formate, pH 5.6, 2% methanol, 4 °C). Afterwards, the CoA-thioesters were analysed using LC‒ESI‒MS/MS. Analyte separation was conducted on a reversed-phase column (Kinetex 2.6 μ XB-C18 100 Å, 100 × 2.1 mm, Phenomenex) at 40 °C with a gradient of eluent A (50 mM formic acid, adjusted to pH 8.1 with 25% ammonium hydroxide) and eluent B (methanol) at a flow rate of 300 μL min^−1^. Multiple reaction monitoring (MRM) was used for the detection of CoA thioesters. Absolute quantification of thioesters was performed as described previously [46]. For absolute quantification of 3-oxoadipyl-CoA, a ^13^C-labelled internal standard was newly obtained from *S. lividans* cells pregrown on 99% 4-hydroxybenzoic acid-[phenyl-^13^C_6_] (Sigma‒Aldrich) [46]. The optimized instrumental settings applied for the analysis of 3-oxoadipyl-CoA were operation in positive ion mode [M + H]^+^ and specific values for the curtain gas (35 psi), collision gas flow rate (medium), ion spray voltage (4.5 kV), temperature (400 °C), ion source gas (60 psi), entrance potential (10 V), mass of the parent ion (*m/z* 910.3) and the daughter ion (*m/z* 403.1), declustering potential (173 V), collision energy (43.2 V) and cell exit potential (24.3 V). The settings for the other CoA esters were taken from previous work [46, 93, 95, 96].

### Transcriptomic analysis

Samples were collected from the production process after 12 h (20,000 ×*g*, 1 min, 4 °C) and immediately frozen in liquid nitrogen. Sample processing, RNA extraction, and RNA sequencing were performed in biological triplicate as described previously [21, 25, 39]. In short, cells (1 mL broth) were collected by centrifugation (20,000 ×*g*, 4 °C, 1 min) and immediately frozen in liquid nitrogen. RNA was extracted with the Qiagen RNA Mini kit (Qiagen, Hilden, Germany) according to the manufacturer’s instructions. Residual DNA was removed by digestion with 10 U RNase-free DNase I (Thermo Scientific) for 1 h in the presence of RiboLock RNase inhibitor (Thermo Scientific). After DNA digestion, the RNA was again purified with the same kit. RNA quality was checked by Trinean Xpose (Gentbrugge, Belgium) and the Agilent RNA 6000 Nano Kit on an Agilent 2100 Bioanalyzer (Agilent Technologies, Böblingen, Germany). Ribosomal RNA (rRNA) molecules were removed from the total RNA with the Ribo-Zero rRNA Removal Kit (Illumina, San Diego, USA). The removal of rRNA was checked with the Agilent RNA 6000 Pico Kit on an Agilent 2100 Bioanalyzer (Agilent Technologies). cDNA libraries were prepared with the TruSeq Stranded mRNA Library Prep Kit (Illumina, San Diego, USA), and the resulting cDNA was paired-end sequenced on an Illumina NextSeq 500 system using a 2 × 75 bp read length. Reads were mapped to the *S. lividans* TK24 ΔYA8-DG2 genome sequence (CP111182.1) with Bowtie2 using standard settings [97], except the maximal allowed distance for paired reads was increased to 600 bases. For visualization of read alignments and raw read count calculation, ReadXplorer 2.2.3 was used [98]. Using the resulting data, DESeq2 [99] was used to QC the datasets via, for example, calculation of the sample-to-sample distances (Additional file 1: Fig. S3) and PCA (Additional file 1: Fig. S2). In addition, DESeq2 was used to calculate the DGE datasets. The raw datasets (sequenced reads) as well as processed datasets (input matrix & normalized read counts from DESeq2) are available from GEO (GSE246798). For statistical analysis, Student’s t test was carried out, and the data were filtered for genes with a log_2_-fold change ≥ 1 (p value ≤ 0.05). Hierarchical clustering was conducted using the software package gplots [100, 101]. RNA extraction and sequencing were conducted in biological triplicates.

### Data processing and statistical analysis

All results displayed in Figures and Tables are shown as the mean values ± standard deviations (SD). Statistical evaluation of the data was conducted by one-way analysis of variance (ANOVA) [102]. Statistical analyses were performed by using SPSS (version 24.0).

### Supplementary Information

**Additional file 1: Table S1.** BLAST analysis of amino acid sequences of genes responsible for the ethylmalonyl-CoA pathway in *S. lividans* ΔYA8-DG2 against *S. venezuelae* ATCC 15439. **Table S2.** Primers for genetic engineering and sequencing. **Table S3.** Chromatographic and mass spectrometric settings during LC–ESI–MS analysis. The conditions for the analysis of bottromycins [20] and pamamycins [39] were adapted from previous work. **Figure S1.** Substrate screening of S. lividans TK24. The minimal plate medium used contained 10 g L−1 mannitol (**A**), 5 mM protocatechuic acid (**B**), 5 mM 4-hydroxybenzoic acid (**C**) as the sole source of carbon, or no carbon source (**D**). The plates were incubated for 5 days at 28 °C. The different concentrations used were chosen based on the expected toxicity of the aromatics. **Figure S2.** Quality assessment of RNA sequencing using PCA. The data comprise the global transcriptomes of *S. lividans* ΔYA8-DG2 grown on mannitol (10 mM), protocatechuate (10 mM), or 4-hydroxybenzoate (10 mM) and sampled after 12 h, n = 3. **Figure S3.** Sample distance plot. The data originate from RNA sequencing of *S. lividans* ΔYA8-DG2 grown on mannitol (10 mM), protocatechuate (10 mM), or 4-hydroxybenzoate (10 mM) and sampled after 12 h, n = 3. **Figure S4.** Volcano plot. The data reflect the differences in gene expression between mannitol- and protocatechuate-grown *S. lividans* ΔYA8-DG2 sampled after 12 h, n = 3. **Figure S5.** Volcano plot. The data reflect the differences in gene expression between mannitol- and 4-hydroxybenzoate-grown *S. lividans* ΔYA8-DG2 sampled after 12 h, n = 3. **Figure S6.** Correlation between optical density and cell dry weight for *S. lividans* ΔYA8-DG2. The strain was grown on 10 mM protocatechuate (**A**) and 30 mM mannitol (**B**), and parallel measurements at different culture time points were performed, n = 3.

**Additional file 2. **LC-MS analysis of bottromycin A2 and methylated bottromycin A2.

**Additional file 3.** Gene expression data reflecting genes linked to amino acid biosynthesis.

**Additional file 4.** Gene expression data reflecting genes linked to oxidative stress response and energy metabolism.

## Data Availability

All dataset(s) supporting the conclusions of this article are included within the article.
