# Supplementary material for: From waste to health-supporting molecules: biosynthesis of natural products from lignin-, plastic- and seaweed-based monomers using metabolically engineered Streptomyces lividans
Source: Microb Cell Fact. 2023 Dec 19;22:262. doi: 10.1186/s12934-023-02266-0 (PMC10731712; doi:10.1186/s12934-023-02266-0)
Supplement: Supplementary file 1 — Additional file 1: Table S1. BLAST analysis of amino acid sequences of genes responsible for the ethylmalonyl-CoA pathway in S. lividans ΔYA8-DG2 against S. venezuelae ATCC 15439. Table S2. Primers for genetic engineering and sequencing. Table S3. Chromatographic and mass spectrometric settings during LC–ESI–MS analysis. The conditions for the analysis of bottromycins [20] and pamamycins [39] were adapted from previous work. Figure S1. Substrate screening of S. lividans TK24. The minimal plate medium used contained 10 g L−1 mannitol (A), 5 mM protocatechuic acid (B), 5 mM 4-hydroxybenzoic acid (C) as the sole source of carbon, or no carbon source (D). The plates were incubated for 5 days at 28 °C. The different concentrations used were chosen based on the expected toxicity of the aromatics. Figure S2. Quality assessment of RNA sequencing using PCA. The data comprise the global transcriptomes of S. lividans ΔYA8-DG2 grown on mannitol (10 mM), protocatechuate (10 mM), or 4-hydroxybenzoate (10 mM) and sampled after 12 h, n = 3. Figure S3. Sample distance plot. The data originate from RNA sequencing of S. lividans ΔYA8-DG2 grown on mannitol (10 mM), protocatechuate (10 mM), or 4-hydroxybenzoate (10 mM) and sampled after 12 h, n = 3. Figure S4. Volcano plot. The data reflect the differences in gene expression between mannitol- and protocatechuate-grown S. lividans ΔYA8-DG2 sampled after 12 h, n = 3. Figure S5. Volcano plot. The data reflect the differences in gene expression between mannitol- and 4-hydroxybenzoate-grown S. lividans ΔYA8-DG2 sampled after 12 h, n = 3. Figure S6. Correlation between optical density and cell dry weight for S. lividans ΔYA8-DG2. The strain was grown on 10 mM protocatechuate (A) and 30 mM mannitol (B), and parallel measurements at different culture time points were performed, n = 3. [file 12934_2023_2266_MOESM1_ESM.docx]

**Table S1. BLAST analysis of amino acid sequences of genes responsible for the ethylmalonyl-CoA pathway in *S. lividans* ΔYA8-DG2 *against S. venezuelae* ATCC 15439.**

| **Gene** | **Annotation** | **Reference** | **Coverage (%) / Identity (%)** |
| --- | --- | --- | --- |
| SLYA8N_06050 | Oxidoreductase | Hydroxybutyryl-CoA dehydrogenase | 99 / 84.71 |
| SLYA8N_06060 | Crotonyl-CoA reductase | Crotonyl-CoA carboxylase | 99 / 91.01 |
| SLYA8N_06065 | Protein MeaA | Ethylmalonyl-CoA mutase | 100 / 87.91 |
| SLYA8N_06070 | Citrate lyase | L-Malyl-CoA lyase | 93 / 92.74 |
| SLYA8N_06075 | MaoC hydratase |  |  |
| SLYA8N_06080 | Acyl-CoA dehydrogenase | Methylsuccinyl-CoA dehydrogenase | 100 / 95.01 |

**Table S2. Primers for genetic engineering and sequencing.**

| **Primers** | **Sequence (5’→3’)** | **Purpose** |
| --- | --- | --- |
| BotAC_fwd | ATGGGACCCGTAGTCGTATTCG | Verification for bottromycin cluster |
| BotAC_rev | TCAGGCGGAACGTCGTCCTT | Verification for bottromycin cluster |
| PamN_fwd | CGAGCACACCCTTCGAGGAA | Verification for pamamycin cluster |
| PamN_rev | GACGGCGTACACGCGGTC | Verification for pamamycin cluster |
| pcaI_fwd | CTTTAAGAAGGAGATATACATATGGCCGGACTGGACAAG | Amplification of *pcaI* sequence |
| pcaI_rev | GCTCGAGTGCGGCCGCAAGCTTGGCCTTGACGGTGCG | Amplification of *pcaI* sequence |
| pcaJ_fwd | CTTTAAGAAGGAGATATACATATGGCACTGACCCGCGAAC | Amplification of *pcaJ* sequence |
| pcaJ_rev | GCTCGAGTGCGGCCGCAAGCTTGAAGGTCATTTCCTTC | Amplification of *pcaJ* sequence |

**Table S3. Chromatographic and mass spectrometric settings during LC-ESI-MS analysis.** The conditions for the analysis of bottromycins [[1](#_ENREF_1)] and pamamycins [[2](#_ENREF_2)] were adapted from previous work.




**Fig. S1: Substrate screening of *S. lividans* TK24**. The minimal plate medium used contained 10 g L^-1^ mannitol (A), 5 mM protocatechuic acid (B), 5 mM 4-hydroxybenzoic acid (C) as the sole source of carbon, or no carbon source (D). The plates were incubated for 5 days at 28 °C. The different concentrations used were chosen based on the expected toxicity of the aromatics [[3](#_ENREF_3)].


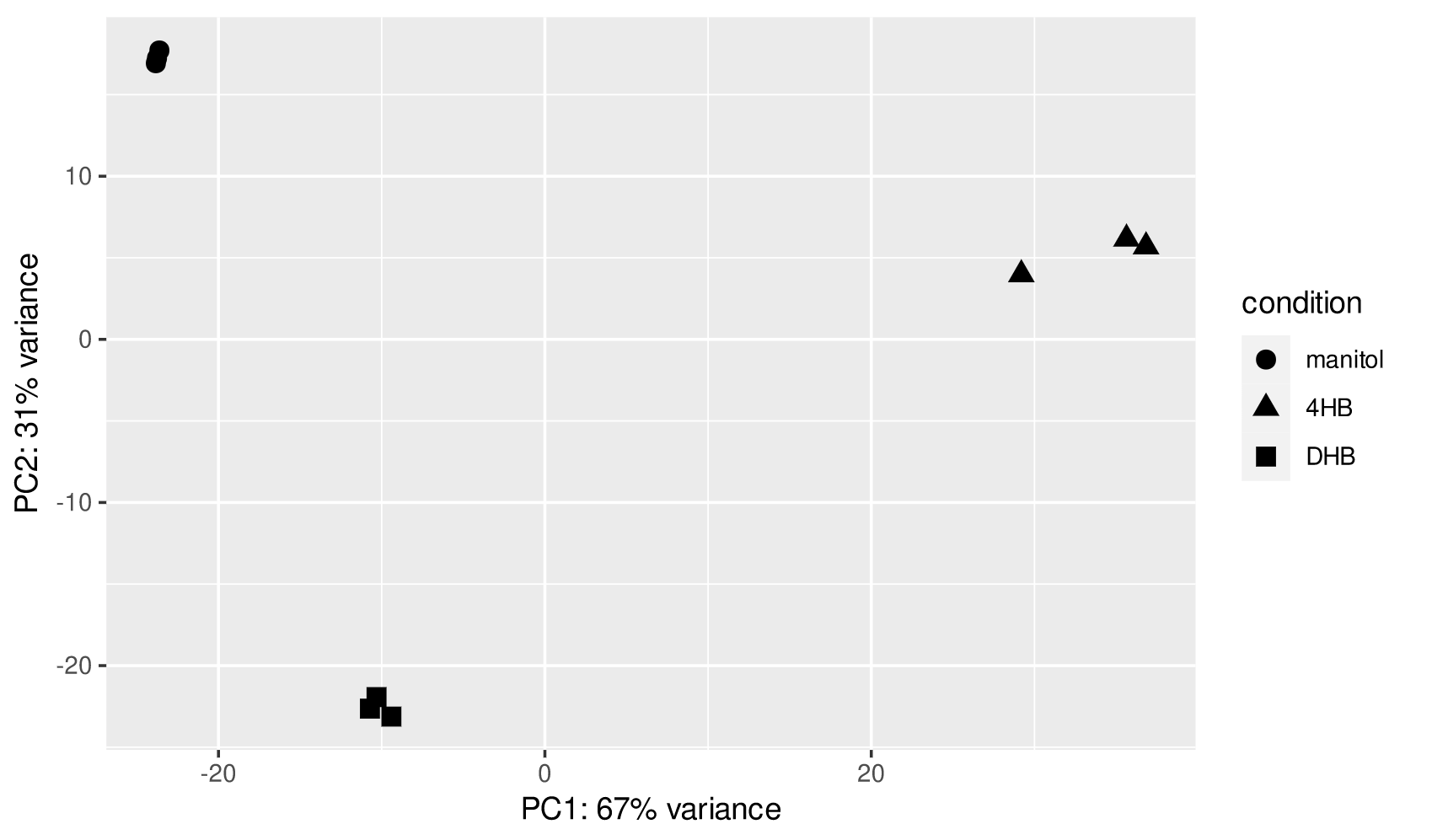


**Figure S2: Quality assessment of RNA sequencing using PCA.** The data comprise the global transcriptomes of *S. lividans* ΔYA8-DG2, grown on mannitol (10 mM), protocatechuate (10 mM), or 4-hydroxybenzoate (10 mM) and sampled after 12 h. n=3.


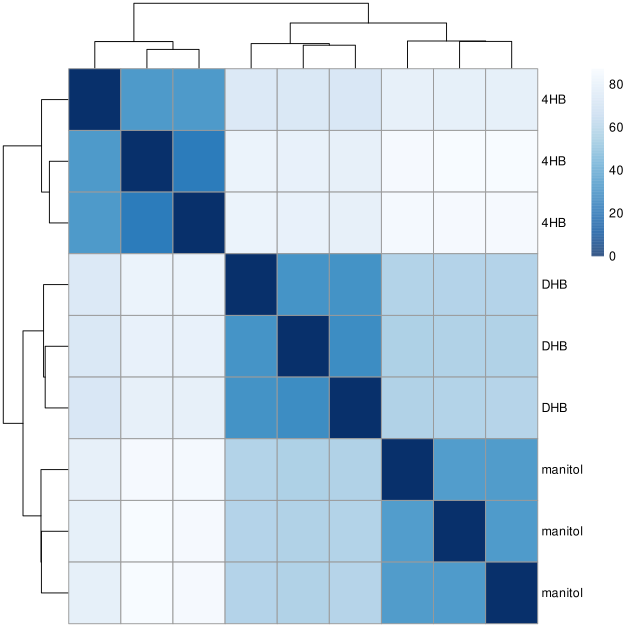


**Figure S3: Sample distance plot.** The data originate from RNA sequencing of *S. lividans* ΔYA8-DG2, grown on mannitol (10 mM), protocatechuate (10 mM), or 4-hydroxybenzoate (10 mM) and sampled after 12 h. n=3.

**
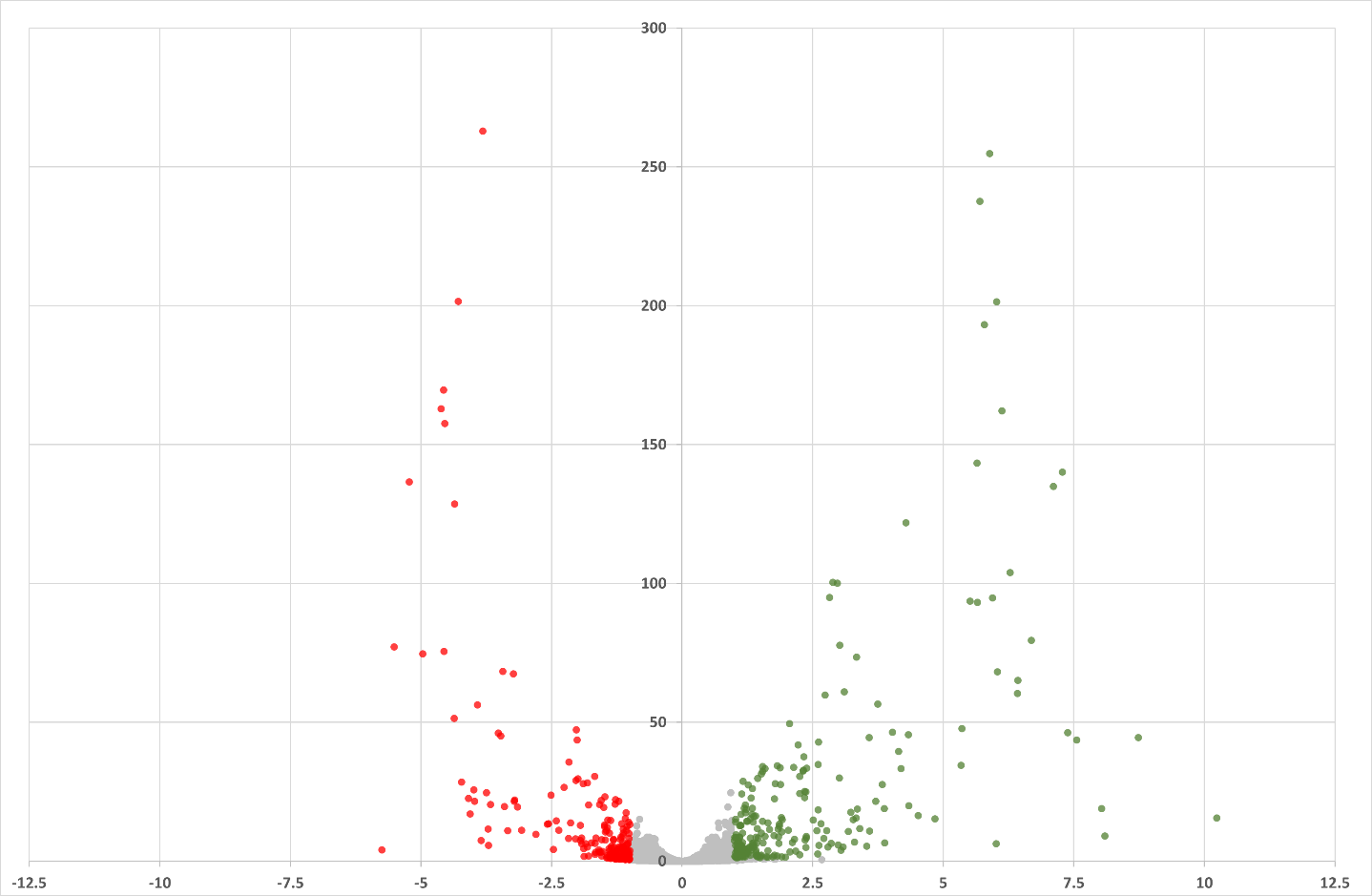
**

**Figure S4: Volcano plot.** The data reflect the differences in gene expression between mannitol- and protocatechuate-grown *S. lividans* ΔYA8-DG2 sampled after 12 h. n=3.

**
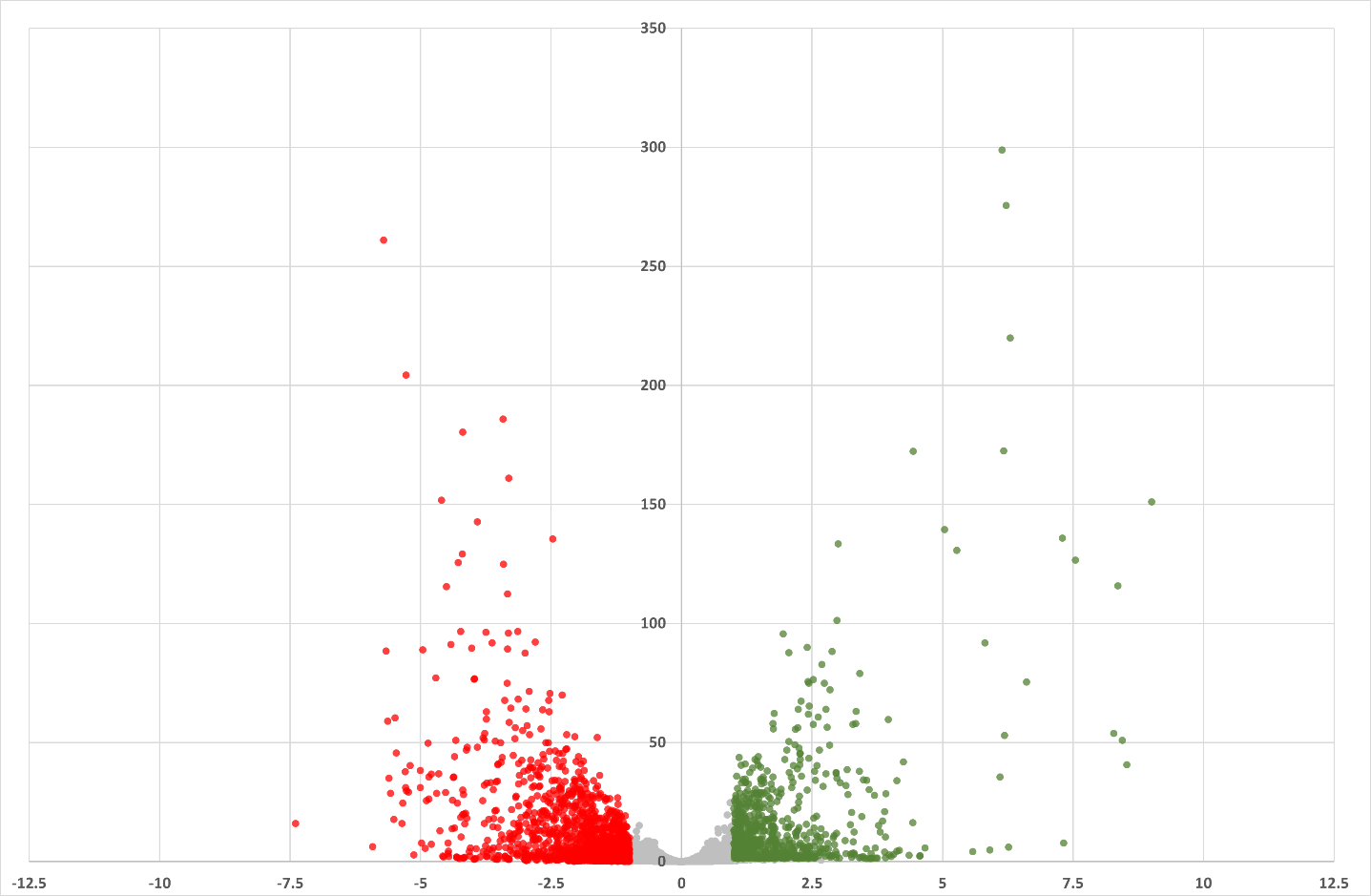
**

**Figure S5: Volcano plot.** The data reflect the differences in gene expression between mannitol- and 4-hydroxybenzoate-grown *S. lividans* ΔYA8-DG2 sampled after 12 h. n=3.

**Figure S6: Correlation between optical density and cell dry weight for *S. lividans* ΔYA8-DG2.** The strain was grown on 10 mM protocatechuate (A) and 30 mM mannitol (B), and parallel measurements at different culture time points were performed. n=3.

**Reference**

1. Horbal L, Marques F, Nadmid S, Mendes MV, Luzhetskyy A: **Secondary metabolites overproduction through transcriptional gene cluster refactoring.** *Metab Eng* 2018, **49:**299-315.

2. Kuhl M, Glaser L, Rebets Y, Ruckert C, Sarkar N, Hartsch T, Kalinowski J, Luzhetskyy A, Wittmann C: **Microparticles globally reprogram *Streptomyces albus* toward accelerated morphogenesis, streamlined carbon core metabolism, and enhanced production of the antituberculosis polyketide pamamycin.** *Biotechnol Bioeng* 2020, **117:**3858-3875.

3. Cho JY, Moon JH, Seong KY, Park KH: **Antimicrobial activity of 4-hydroxybenzoic acid and 4-hydroxycinnamic acid isolated and identified from rice hull.** *Bioscience Biotechnology and Biochemistry* 1998, **62:**2273-2276.
